# Efficient, highly diastereoselective MS 4 Å-promoted one-pot, three-component synthesis of 2,6-disubstituted-4-tosyloxytetrahydropyrans via Prins cyclization

**DOI:** 10.3762/bjoc.8.19

**Published:** 2012-02-01

**Authors:** Naseem Ahmed, Naveen Kumar Konduru

**Affiliations:** 1 Department of Chemistry, Indian Institute of Technology Roorkee, Roorkee- 247 667, Uttarakhand, India

**Keywords:** aromatic homoallylic alcohols, 2,6-disubstituted-4-tosyloxytetrahydropyrans, MS 4 Å, Prins cyclization, PTSA

## Abstract

A simple, efficient and highly diastereoselective one-pot three-component synthesis of functionalized 2,6-disubstituted-4-tosyloxytetrahydropyrans was performed. The synthesis features an optimized Prins cyclization in which an aromatic homoallylic alcohol, an aromatic/aliphatic aldehyde, and *p*-toluenesulfonic acid (catalyst and reagent) are reacted in the presence of molecular sieves (MS) 4 Å at reflux in dichloromethane to afford excellent yields (72–96%) within short reaction times (20–90 min). The MS 4 Å-promoted synthesis proved to be versatile enough to provide an array of symmetrical and unsymmetrical tetrahydropyran derivatives in economical manner. Furthermore, cleavage of the 4-tosyl group under mild conditions afforded 4-hydroxytetrahydropyran in excellent yields (95–96%).

## Introduction

**Figure 1:**
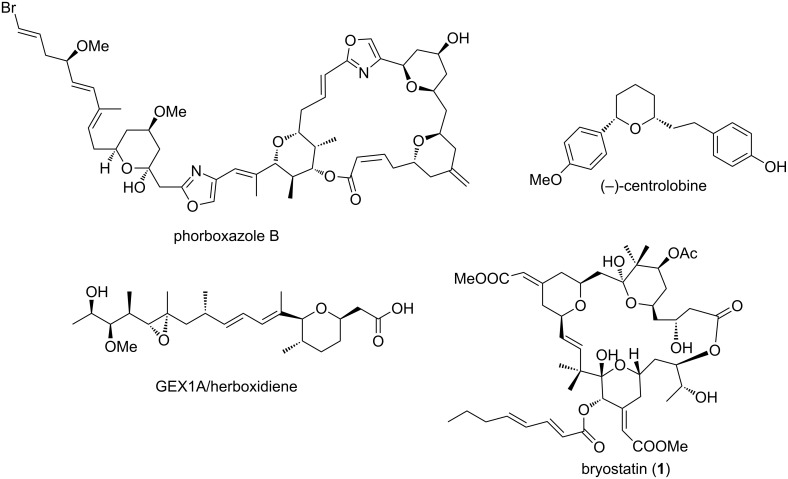
Tetrahydropyran ring containing natural products.

Substituted tetrahydropyrans are common structural motifs in numerous biological molecules and natural products that include phorboxazoles (A and B) [1], (−)-centrolobine [2], GEX1A/herboxidiene [3], bryostatins [4], and pheromones [5] (Figure 1). Tetrahydropyran derivatives are also used as materials in photographic films [6] and host–guest chemistry [7]. In particular, 2,4,6-trisubstituted tetrahydropyrans have tremendous applications in pharmaceuticals and are widely present in biologically active core structures such as 4-oxygenated, 4-halogenated, 4-sulfonyl- and 4-azido/amidotetrahydropyrans [8–11]. Various applications of tetrahydropyran derivatives have inspired organic chemists to develop their efficient, economical and stereoselective synthesis.

To introduce the desired substituents and stereochemistry at the 2, 4 and 6-positions of the tetrahydropyran ring, Prins cyclization has been considered to be the best approach. The reaction can be conducted either with Brønsted acid catalysts – namely TFA [12], AcOH, MeSO_3_H [13], Sc(OTf)_3_ [14], O_3_ReOSiPh_3_ [15], TMSBr [16] – or with Lewis acid catalysts such as AlCl_3_ [17], InCl_3_ [18–19], TiCl_4_, HBF_4_·OEt_2_ [20], InBr_3_ [21], TiBr_4_ [22], Fe(III) compounds [23], BF_3_·OEt_2_ [24], polymer supported [25], microwave accelerated with BiCl_3_ [26], and Pd^0^/Sn^II^ mediated by a three component cascade coupling (3-C^3^) [27]. However, these procedures suffer from drawbacks like moderate yields, high/low reaction temperatures, extended reaction times, and/or strong acid conditions [28–29]. Therefore, Wills et al. reported an efficient synthetic route for 2,4,6**-**trisubstituted tetrahydropyrans [30]; however, this method is restricted to electron withdrawing groups containing aromatic homoallylic alcohols. Similarly, She et al. recently have reported a new approach for the synthesis of 2,4,6-trisubstituted tetrahydropyrans via a Prins cyclization [31]. With this method, both symmetrical and unsymmetrical tetrahydropyrans can be synthesized.

*p-*Toluenesulfonic acid (PTSA) is reported as a versatile Brønsted acid catalyst in various organic transformations [32–34]. Previously, PTSA has been used as a catalyst in Prins cyclizations but the product yields were low even under extended reaction times [10].

In continuation of our interest in acid catalysis [35–39], we report herein an efficient, economical and highly diastereoselective one-pot three-component synthesis for both symmetrical and unsymmetrical 2,4,6-trisubstituted tetrahydropyrans using aromatic homoallyl alcohols, aromatic/aliphatic aldehydes and *p-*toluenesulfonic acid (both as reagent and catalyst) in the presence of molecular sieves (MS 4 Å) in dichloromethane at reflux. The molecular sieves (MS 4 Å) had a significant effect on improving product yields (72–96%) under short reaction times (20–90 min). Further, cleavage of the 4-tosyl group under mild conditions afforded 4-hydroxytetrahydropyran in excellent yields (95–96%).

## Results and Discussion

The starting materials, aromatic homoallylic alcohols, were readily prepared by treatment of aromatic aldehydes with allylic Grignard reagents under a nitrogen atmosphere at −78 °C for 2 h [40]. Their structures were assigned by ^1^H, ^13^C NMR, IR, and GC–MS data and compared with reported values in the literature.

**Scheme 1:**
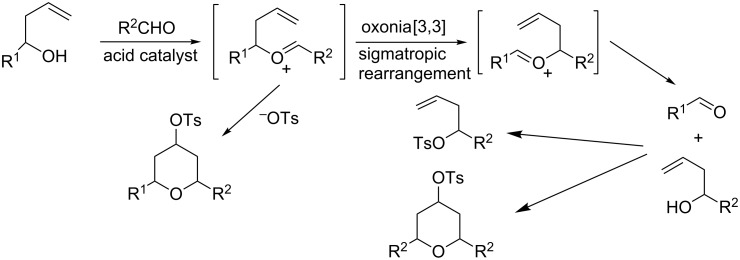
Plausible side products mechanism.

Initially, we carried out the reaction with homoallylic alcohols, aromatic aldehydes and PTSA at room temperature to afford 2,4,6-trisubstituted tetrahydropyrans. After 22 h stirring more side-products than the desired product were observed. This might be due to the formation of an oxo-carbenium intermediate, which further reacted in a [3,3]sigmatropic rearrangement to give another oxo-carbenium ion (Scheme 1).

To optimize the reaction yield, we varied the reaction conditions such as enhancing the catalytic loading (1.2 equiv to 1.4 equiv), varying solvents and temperature, rearranging the order of reagent addition and adding MS 4 Å as drying agent (Table 1).

**Table 1:** Optimization of reaction yields.

Entry^a^	Brønsted acid	Solvent	Temp (°C)	Time	Yield (%)^b^

1	PTSA (1.2 equiv)	Toluene	rt	20 h	25
2	PTSA (1.2 equiv)	EtOAc	rt	20 h	22
3	PTSA (1.2 equiv)	THF	rt	28 h	26
4	PTSA (1.2 equiv)	DCE	rt	18 h	25
5	PTSA (1.2 equiv)	DCM	rt	22 h	30
6	PTSA (1.4 equiv)	DCM	rt	20 h	35
7	PTSA (1.4 equiv)	THF	66	22 h	28
8	PTSA (1.4 equiv)	DCM	rt	40 min	45
9	PTSA (1.4 equiv)	DCM	40	20 min	75
10^c^	**PTSA (1.4 equiv)**	**DCM**	**40**	**20 min**	**94**

^a^All reactions were carried out with homoallylic alcohol (1 equiv), aldehyde (1 equiv) and PTSA (1.2–1.4 equiv). ^b^Isolated yield. ^c^MS 4 Å (30 mg/equiv) was used.

Among different solvents (toluene, THF, DCM, DCE and diethyl ether) that were used, DCM was found as the solvent of choice at reflux temperature (Table 1, entries 5, 6, 8–10). Similarly, various acids like trifluoroacetic acid (TFA), benzoic acid, acetic acid, benzylphosphonic acid, and *O*,*O*’-diethyl dithiophosphate were tried as catalysts but all of them failed to give the desired product. We expected that the order of reagent addition would have significant influence on the yield. For example, the addition of PTSA (1.4 equiv) to a stirred solution of homoallylic alcohol and benzaldehyde in dichloromethane (DCM) at room temperature gave the product in 35% yield (Table 1, entry 6). Compared to that, the addition of homoallylic alcohol to a stirred mixture of PTSA (1.4 equiv) and aromatic aldehyde in DCM improved the yield (45%) at the same temperature (Table 1, entry 8). Following the later addition order at reflux (40 °C) in DCM (Table 1, entry 9), the yield was enhanced up to 75% within 20 min. Furthermore, the addition of molecular sieves (MS 4 Å) to the above mentioned reaction mixture within 20 min resulted in an essential improvement of the yield (94%, Table 1, entry 10). In the presence of MS 4 Å, the yield was unexpectedly enhanced from 75% to 94% under the same reaction conditions (Table 1, entry 9 vs entry 10). The significant improvements in product yields, reaction time and/or diastereoselectivity might be due to the prevention of the [3,3]sigmatropic rearrangement along with the dehydrating activity of MS 4 Å. In addition, we studied the stoichiometric ratio of MS 4 Å with respect to the substrate and found that 30 mg/mmol of MS 4 Å are necessary to receive optimal yields.

Under optimal reaction conditions we reacted a wide selection of aromatic homoallylic alcohols and aldehydes. The experimental results are summarized in Table 2. In all cases, the corresponding tetrahydropyrans were obtained in high diastereoselectivity and excellent yields without side products (Table 2). A high degree of diastereoselectivity was determined from the ^1^H NMR spectra without purification (crude product). We observed that substituents on the aromatic rings influenced the reaction rates and yields. For example, strong electron-donating groups such as methoxy or trimethoxy at homoallylic alcohols afforded the corresponding tetrahydropyrans in lower yields (72–75%) but in a faster rate (Table 2, entries 7 and 9). Similarly, the presence of electron-withdrawing substituents – such as chlorine or bromine atoms at homoallylic alcohols – gave the corresponding tetrahydropyrans in high yields (85–96%) but the reaction times were longer (Table 2, entries 2–4, 6, 8, 10). However, substituents on aldehydes have no significant effect on the reaction time or yields (Table 2, entries 2, 4, 6, 12–15). We further extended our method to aliphatic aldehydes (e.g., acetaldehyde) and hetero aromatic aldehydes (e.g., pyrrole aldehyde, furfural). Under optimal reaction conditions they reacted smoothly with homoallylic alcohols to afford the corresponding tetrahydropyran derivatives which show almost the same distereoselectivity and product yields (83–89%, Table 2, entries 16–18).

**Table 2:** Preparation of tetrahydropyrans from aromatic homoallylic alcohols, aldehydes and PTSA·H_2_O.

Entry	Homoallylic alcohol	Aldehyde	Product	Reaction time (min)	Yield (%)

1	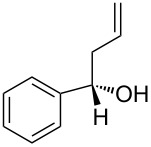 **1a**	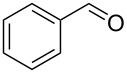	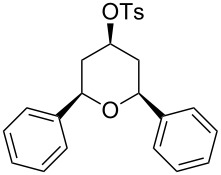 **1b**	90	94
2	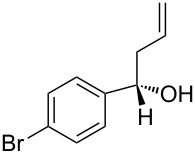 **4a**	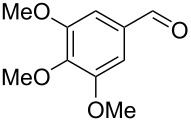	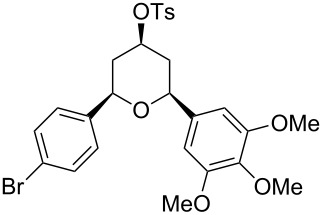 **2b**	20	82
3	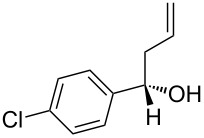 **2a**	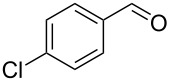	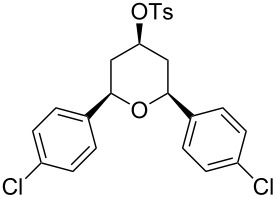 **3b**	90	92
4	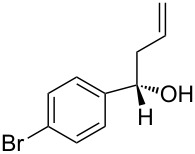 **4a**	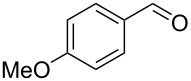	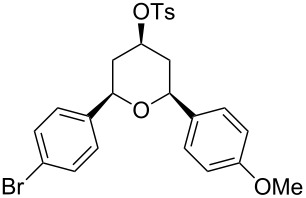 **4b**	25	88
5	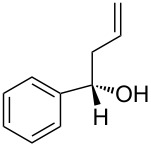 **1a**	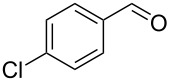	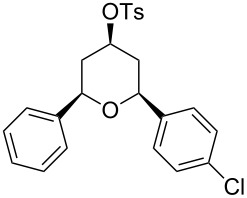 **5b**	90	90
6	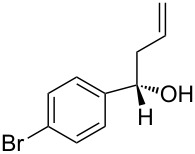 **4a**	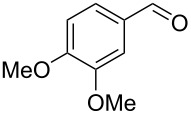	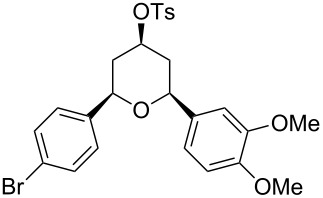 **6b**	35	85
7	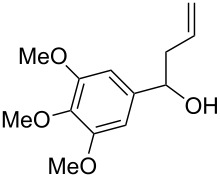 **5a**	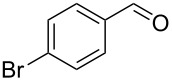	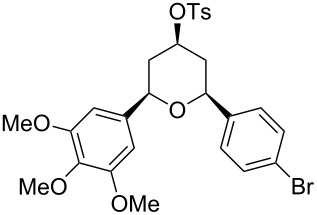 **7b**	23	72
8	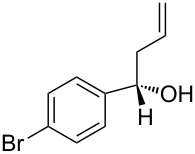 **4a**	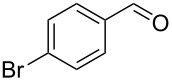	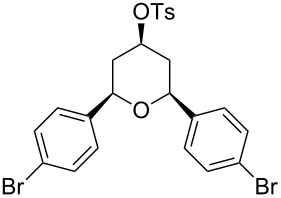 **8b**	90	95
9	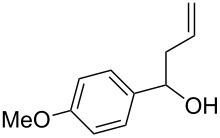 **3a**	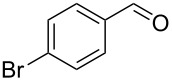	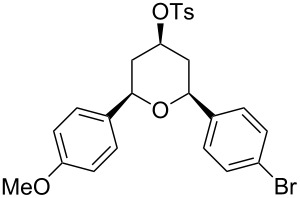 **9b**	25	75
10	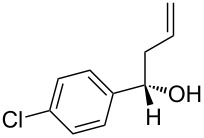 **2a**	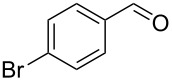	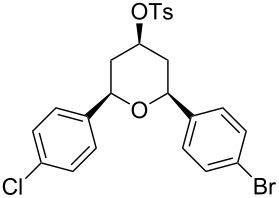 **10b**	90	95
11	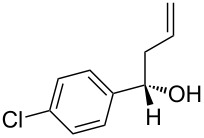 **2a**	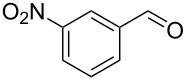	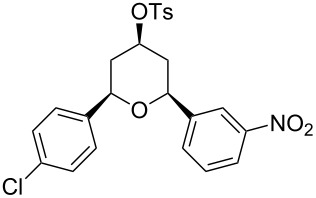 **11b**	90	96
12	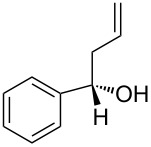 **1a**	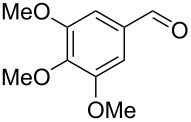	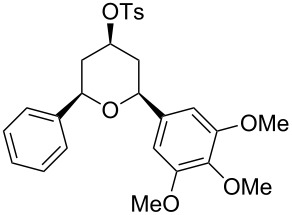 **12b**	20	83
13	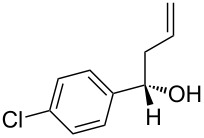 **2a**	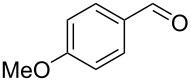	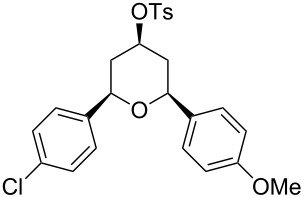 **13b**	30	88
14	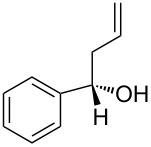 **1a**	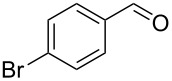	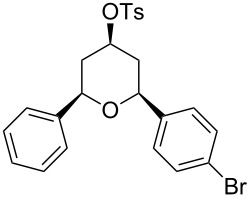 **14b**	90	95
15	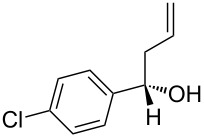 **2a**	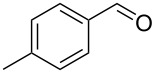	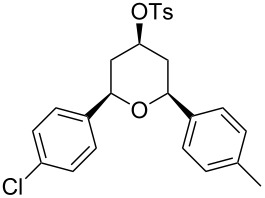 **15b**	60	93
16	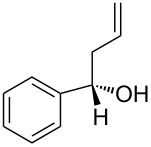 **1a**	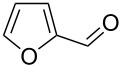	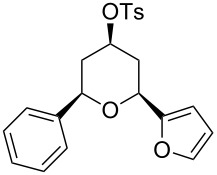 **16b**	50	83
17	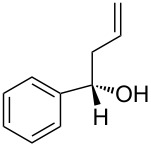 **1a**	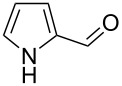	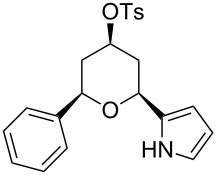 **17b**	50	86
18	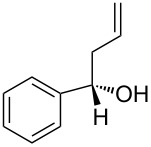 **1a**	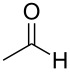	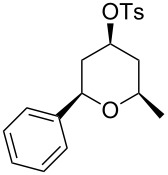 **18b**	75	89

**Scheme 2:**
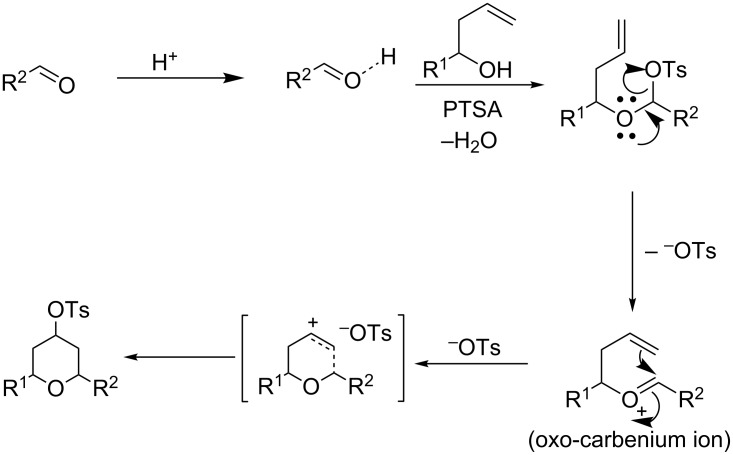
Plausible reaction mechanism via Prins cyclization.

From a mechanistical point of view, these reactions are similar to the Prins cyclization [41]. First, the aldehyde got activated by PTSA protonation followed by a nucleophilic attack of the homoallylic alcohol and proton transfer to the hydroxy group. Then, a nucleophilic attack of PTSA resulted in α-tosyloxyether formation after losing a water molecule. In the α-tosyloxyether, the delocalization of lone-pair electrons on the oxygen atom led to the removal of the tosylate group and oxo-carbenium ion intermediate formation. Then, an intramolecular nucleophilic attack of the double bond in the oxo-carbenium ion led to cyclization and charge transfer complex formation with the tosylate group to afford the 2,6-disubstituted-4-tosyloxytetrahydropyran (Scheme 2).

**Figure 2:**
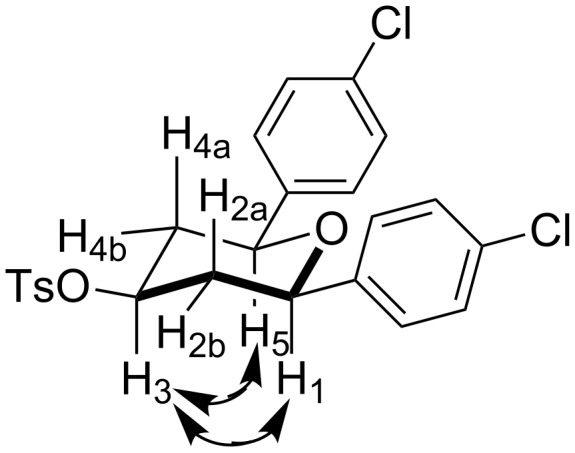
Schematic NOE diagram of compound **3b**.

All structures of the 2,6-disubstituted-4-tosyloxytetrahydropyrans were established by ^1^H, ^13^C NMR, IR, and GC–MS spectral data and elemental analysis. NOE studies of compound **3b** (Table 2, entry 3) confirmed that all three substituents occupy equatorial positions on the tetrahydropyran ring. The NOE cross peaks H_1_/H_3_, H_5_/H_3_ reveal that H_1,_ H_5_ and H_3_ are on the same side of the ring and take a diaxial orientation (Figure 2). ^1^H NMR experiments were performed at 500 MHz in CDCl_3_. The proposed structure of **3b** (Table 1, entry 3) is further supported by the coupling constants *J*_H1/H2b_ = 1.5 Hz, *J*_H1/H2a_ = 11.5 Hz, *J*_H2b/H3_ = 4.5 Hz and *J*_H2a/H3_ = 11.5 Hz. Thus, H_2a_ and H_4a_ are antiperiplanar to H_1,_ H_3_ and H_5_. All these findings confirm that the pyran ring takes a chair conformation, where the substituents at C_1_, C_3_ and C_5_ are present in equatorial position.

**Scheme 3:**
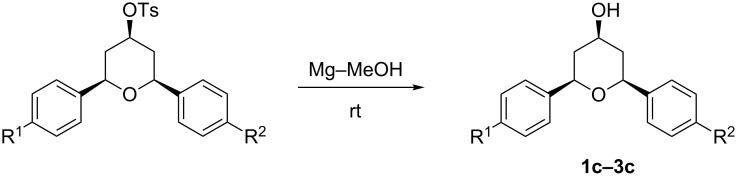
Deprotection of the hydroxy group.

The tosyl group at C_4_ got easily deprotected at room temperature with Mg–MeOH (Scheme 3) [42] to afford 2,6-disubstited-4-hydroxytetrahydropyrans with retention of the stereochemistry in quantitative yield (Table 3). The structures of 2,6-disubstituted-4-hydroxytetrahydropyrans were established by ^1^H, ^13^C NMR, IR, and GC–MS and elemental analysis and were compared with reported data [43].

**Table 3:** Preparation of 2,6-disubstituted-4-hydroxytetrahydropyrans.

Entry	4-Tosyloxytetrahydropyran (R^1^, R^2^)	4-Hydroxytetrahydropyran (R^1^, R^2^)	Yield (%)

1	Cl, Cl	Cl, Cl	**1c** (96)
2	H, H	H, H	**2c** (95)
3	Br, Br	Br, Br	**3c** (95)

## Conclusion

In conclusion, we have reported a simple and efficient one-pot three-component synthesis of highly diastereoselective and functionalized 2,6-disubstituted-4-tosyloxytetrahydropyrans via Prins cyclization. An aromatic homoallylic alcohol, an aromatic/aliphatic aldehyde, and *p*-toluenesulfonic acid (catalyst and reagent) are reacted in the presence of MS 4 Å in dichloromethane at reflux to afford 2,6-disubstituted-4-tosyloxytetrahydropyrans in excellent yields (72–96%) within short reaction times (20–90 min). The MS 4 Å promoted synthesis proved to be versatile enough to provide an array of symmetrical and unsymmetrical tetrahydropyran derivatives in an economical manner. Moreover, it was observed that MS 4 Å might have a vital part in controlling the reversibility of the [3,3]sigmatropic rearrangement. Furthermore, cleavage of the 4-tosyl group under mild conditions afforded 4-hydroxytetrahydropyrans in high diastereoselectivity and excellent yields (95–96%).

## Supporting Information

File 1 Experimental details and characterization data of synthesized compounds, ^1^H and ^13^C NMR spectra.
