# Supplementary material for: Efficient, highly diastereoselective MS 4 Å-promoted one-pot, three-component synthesis of 2,6-disubstituted-4-tosyloxytetrahydropyrans via Prins cyclization
Source: Beilstein J Org Chem. 2012 Feb 1;8:177–85. doi: 10.3762/bjoc.8.19 (PMC3302078; doi:10.3762/bjoc.8.19)
Supplement: File 1 — Experimental details and characterization data of synthesized compounds, 1H and 13C NMR spectra. [file Beilstein_J_Org_Chem-08-177-s001.pdf]

# Supporting Information

# for

# Efficient, highly diastereoselective MS 4 Å-promoted one-pot, three-component synthesis of 2,6-disubstituted-4-tosyloxytetrahydropyrans via Prins cyclization

Naseem Ahmed* and Naveen Kumar Konduru

Address: Department of Chemistry, Indian Institute of Technology Roorkee, Roorkee- 247 667, Uttarakhand, India

Email: Naseem Ahmed*- nasemfcy@iitr.ernet.in
* Corresponding author

## Experimental details and characterization data of synthesized compounds, $^{1}H$ and $^{13}C$ NMR spectra

### Table of contents

1. General methods S2
2. General procedure for the synthesis of 4-tosyloxy tetrahydropyrans (**1b**–**18b**) S2
3. General procedure for the synthesis of 4-hydroxy tetrahydropyrans (**1c**–**3c**) S2
4. General procedure for the preparation of homoallylic alcohols (**1a**–**5a**) S3
5. Characterization data for 4-tosyloxy-, 4-hydroxytetrahydropyrans and homoallylic alcohols S3–S13
6. $^{1}H$ NMR and $^{13}C$ NMR spectra for 4-tosyloxy- and 4-hydroxytetrahydropyrans S14–S31

## 1. General methods

Organic solvents were dried by standard methods, the reagents (chemicals) were purchased from commercial sources, and used without further purification. All reactions were monitored by TLC using precoated silica gel aluminum plates. Visualization of TLC plates was accomplished with an UV lamp. Column chromatography was performed using silica gel 100–200 mesh size (SD Fine-Chem Limited) with EtOAc–hexanes as eluent. Melting points were recorded on Perfit apparatus and are uncorrected. All products were characterized by NMR, IR and MS spectra. $^{1}$H and $^{13}$C NMR spectra were recorded in deuterated chloroform ($CDCl_3$) on a 500 MHz and 125 MHz spectrometer (Bruker), respectively. Chemical shifts were reported in parts per million (ppm, δ) downfield from tetramethylsilane. Proton coupling patterns are described as singlet (s), doublet (d), triplet (t), quartet (q), triplet of triplet (tt), multiplet (m), and broad (br). Elemental analysis was performed by using Perkin-Elmer 2400 series CHNS/O analyzer.

**2. General procedure for the synthesis of 4-tosyloxytetrahydropyrans:** Aldehyde (1 mmol) and *p*-toluenesulfonic acid (PTSA, 1.4 mmol) were dissolved in dichloromethane (2 mL) and stirred for 5 min at 40 °C. Then, aromatic homoallylic alcohol (1 mmol, dissolved in 2 mL DCM) followed by MS 4 Å were added to the stirred reaction mixture. Furthermore, the reaction mixture was stirred at the same temperature for a specified time (Table 2). TLC monitoring, after completion of the reaction the reaction mixture was cooled and aq. $NaHCO_3$ solution was added. The product was extracted with dichloromethane (3 x 10 mL). The combined organic layer was washed with brine (5 mL) and water (5 mL) followed by drying with anhyd. $Na_2SO_4$. After evaporation of the solvent in vacuo, the pure product was obtained. In some cases, purification by silica gel column chromatography using hexane:EtOAc as eluent was necessary.

**3. General procedure for the synthesis of 4-hydroxytetrahydropyrans from toluenesulfonates using Mg–MeOH:** 4-Tosyloxytetrahydropyran (0.5 mmol) and magnesium (120 mg, 5 mmol) in dry methanol (5 mL) in a round bottom flask fitted with a condenser and calcium chloride guard tube was stirred at room temperature with a keeping flask in a water bath for 6 h. After the reaction was complete, the reaction mixture was neutralized with chilled 5% HCl and extracted with diethyl ether (3 x 8 mL).The combined organic layers were washed with water, brine and dried over anhydrous $Na_2SO_4$ and concentrated. Crystallization from ethanol yielded pure tetrahydropyran-4-ol.

**4. General procedure for the preparation of homoallylic alcohols:** To a cooled (–78 °C) solution of aromatic aldehyde (5 mmol) in THF (30 mL) was added $CH_2$=$CHCH_2MgBr$ (10 mL, 1.0 M in THF, 10 mmol). After the reaction mixture was stirred for 2 h at the same temperature, it was quenched with saturated aqueous $NH_4Cl$ and diluted with EtOAc. The layers were separated and the aqueous layer was extracted with EtOAc. The combined organic layers were washed with brine, dried over anhydrous $Na_2SO_4$, and concentrated in vacuo. The residue was purified by column chromatography (silica gel, hexanes/EtOAc, 10/1 v/v) to afford the known homoallylic alcohol.

**5. Characterization data for 4-tosyloxytetrahydropyrans, 4-hydroxytetrahydropyrans and homoallylic alcohols:**

**(2*R*,4*s*,6*S*)-2,6-diphenyl-4-tosyloxytetrahydropyran (1b)**

Yellow solid; yield: 376 mg (92%); mp 100-102 °C. $^1$H NMR (500 MHz, $CDCl_3$): δ = 7.88 (d, *J* = 8 Hz, 2 H, Ar-H), 7.43-7.37 (m, 10 H, Ar-H), 7.34-7.31 (m, 2 H, Ar-H), 5.03 (tt, *J* = 4.5 and 11.5 Hz, 1 H, H-C-OTs), 4.59 (d, *J* = 11.5 Hz, 2 H, Ar-CH-O), 2.48 (s, 3 H, Me), 2.34 (dd, *J* = 4.5 and 12.5 Hz, 2 H, C-$CH_{eq}$-C), 1.90 (q, *J* = 11.5 Hz, 2 H, C-$CH_{ax}$-C). $^{13}$C NMR (125 MHz,

$CDCl_3$): δ = 144.75, 140.91, 134.20, 129.84, 128.34, 127.75, 127.5, 125.78, 78.18, 77.41, 39.94, 21.56. IR (KBr): 3056, 3039, 2923, 2852, 2373, 1717, 1629, 1454, 1379, 1178, 1065, 945, 903, 757, 699 $cm^{-1}$. Anal. Calcd for $C_{24}H_{24}O_4S$ (408.5): C, 70.56; H, 5.92; S, 7.85. Found: C, 70.49; H, 5.91; S, 7.68.

**(2*R*,4*R*,6*S*)-2-(4-bromophenyl)-6-(3,4,5-trimethoxyphenyl)-4-tosyloxytetrahydropyran (2b)**

Yellow semisolid; yield: 473.5 mg (82%). $^1$H NMR (500 MHz, $CDCl_3$): δ = 7.81 (d, *J* = 8.5 Hz, 2 H, Ar-H), 7.45 (d, *J* = 8.5 Hz, 2 H, Ar-H), 7.34 (d, *J* = 8.5 Hz, 2 H, Ar-H), 7.22 (d, *J* = 8.5 Hz, 2 H, Ar-H), 6.56 (s, 2 H, Ar-H), 4.94 (tt, *J* = 5.0 and 11.0 Hz, 1 H, H-C-OTs), 4.50-4.44 (m, 2 H, Ar-CH-O), 3.85 (s, 6 H, OMe), 3.81 (s, 3 H, OMe), 2.44 (s, 3 H, Me), 2.32-2.25 (m, 2 H, C-$CH_{eq}$-C), 1.85 (q, *J* = 11.5 Hz, 1 H, C-$CH_{ax}$-C), 1.79 (q, *J* = 11.5 Hz, 1 H, C-$CH_{ax}$-C). $^{13}$C NMR (125 MHz, $CDCl_3$): δ = 153.31, 144.96, 139.84, 137.73, 136.29, 134.23, 131.61, 129.96, 127.62, 121.77, 103.15, 77.89, 77.73, 71.81, 60.84, 56.18, 39.82, 21.7. IR (KBr): 2960, 2925, 2843, 1726, 1594, 1461, 1382, 1175, 1126, 1010, 953, 904, 817 $cm^{-1}$. Anal. Calcd for $C_{27}H_{29}BrO_7S$ (577.48): C, 56.16; H, 5.06; S, 5.55. Found: C, 56. 03; H, 5.49; S, 5.65.

**(2*R*,4*s*,6*S*)-2,6-bis(4-chlorophenyl)-4-tosyloxytetrahydropyran (3b)**

Yellow solid; yield: 439.2 mg (92%); mp 106-108 $^o$C. $^1$H NMR (500 MHz, $CDCl_3$): δ = 7.81 (d, *J* = 8 Hz, 2 H, Ar-H), 7.35-7.26 (m, 10 H, Ar-H), 4.93 (tt, *J* = 4.5 and 11.5 Hz, 1 H, H-C-OTs), 4.50 (dd, *J* = 1.5 and 11.5 Hz, 2 H, Ar-CH-O), 2.44 (s, 3 H, Me), 2.27 (dd, *J* = 4.5 and 11.5 Hz, 2 H, C-$CH_{eq}$-C), 1.75 (q, *J* = 11.5 Hz, 2 H, C-$CH_{ax}$-C). $^{13}$C NMR (125 MHz, $CDCl_3$): δ = 145.0, 139.3, 134.2, 133.7, 130.0, 128.7, 127.6, 127.2, 77.6, 76.9, 39.9, 21.7. IR (KBr): 3051, 2956, 2924, 2853, 1721, 1634, 1596, 1491, 1353, 1293, 1171, 1089, 1014, 950, 908, 859, 828, 665 $cm^{-1}$. Anal. Calcd for $C_{24}H_{22}Cl_2O_4S$ (477.4): C, 60.38; H, 4.64; S, 6.72. Found: C, 60.45; H, 4. 65; S, 6.55.

**(2*R*,4*S*,6*S*)-2-(4-bromophenyl)-6-(4-methoxyphenyl)-4-tosyloxytetrahydropyran (4b)**

yellow semisolid; yield: 455.3 mg (88%). $^1$H NMR (500 MHz, $CDCl_3$): δ = 7.81 (d, *J* = 8.5 Hz, 2 H, Ar-H), 7.45 (d, *J* = 8.5 Hz, 2 H, Ar-H), 7.34 (d, *J* = 8.5 Hz, 2 H, Ar-H), 7.27 (d, *J* = 9 Hz, 2 H, Ar-H), 7.22 (d, *J* = 8.5 Hz, 2 H, Ar-H), 6.87 (d, *J* = 9 Hz, 2 H, Ar-H), 4.92 (tt, *J* = 4.5 and 11.5 Hz, 1 H, H-C-OTs), 4.48 (t, *J* = 10 Hz, 2 H, Ar-CH-O), 3.79 (s, 3 H, OMe), 2.45(s, 3 H, Me), 2.29-2.22 (m, 2 H, C-CH-$C_{eq}$), 1.83 (q, *J* = 11.5 Hz, 1 H, C-$CH_{ax}$-C), 1.76 (q, *J* = 11.5 Hz, 1 H, C-$CH_{ax}$-C). $^{13}$C NMR (125 MHz, $CDCl_3$): δ = 158.2, 143.8, 139.0, 133.2, 131.8, 130.5, 128.9, 126.5, 126.2, 120.6, 112.8, 76.9, 75.7, 54.2, 38.9, 38.7, 20.6. IR (KBr): 2920, 2850, 1631, 1499, 1383, 1299, 1248, 1175, 1073, 904, 813, 730, 721, 669 cm$^{-1}$. Anal. Calcd for $C_{25}H_{25}BrO_5S$ (517.43): C, 58.03; H, 4.87; S, 6.20. Found: C, 58.11; H, 4.90; S, 6.28.

**(2*S*,4*R*,6*R*)-2-(4-chlorophenyl)-6-phenyl-4-tosyloxytetrahydropyran (5b)**

Yellow solid; yield: 398.65 mg (90%); mp 95-97 °C. $^1$H NMR (500 MHz, $CDCl_3$): δ = 7.82.(dd, *J* = 2 and 8.5 Hz, Ar-H), 7.38-7.261 (m, 10 H, Ar-H), 4.96 (tt, *J* = 5.5 and 11 Hz, 1 H, H-C-OTs), 4.55-4.50 (m, 2 H, Ar-CH-O), 2.48 (s, 3 H, Me), 2.30 (m, 2 H, C-$CH_{eq}$-C), 1.85 (m, 2 H, C-$CH_{ax}$-C). $^{13}$C NMR (125 MHz, $CDCl_3$): δ = 144.9, 140.9, 139.4, 134.3, 133.5, 129.9, 128.6, 128.4, 128.0, 127.6, 127.2, 125.9, 78.0, 77.6, 77.6, 40.0, 39.9, 21.7. IR (KBr): 3056, 2962, 1600, 1149, 1356, 1261, 1172, 1095, 1022, 802, 560 cm$^{-1}$. Anal. Calcd for $C_{24}H_{23}ClO_4S$ (442.95): C, 65.08; H, 5.23; S, 7.24. Found: C, 64.18; H, 5.25; S, 7.30.

**(2*R*,4*R*,6*S*)-2-(4-bromophenyl)-6-(3,4-dimethoxyphenyl)-4-tosyloxytetrahydropyran (6b)**

Yellow solid; yield: 465.33 mg (85%); mp 111-113 °C. $^1$H NMR (500 MHz, $CDCl_3$): δ = 7.81 (d, *J* = 8 Hz, 2 H, Ar-H), 7.45 (d, *J* = 8 Hz, 2 H, Ar-H), 7.34 (d, *J* = 8 Hz, 2 H, Ar-H), 7.22 (d, *J* = 8.5 Hz, 2 H, Ar-H), 6.90-6.82 (m, 3 H, Ar-H), 4.93 (tt, *J* = 4.5 and 11.5 Hz, 1 H, H-C-OTs), 4.48 (t, *J* = 11.5 Hz, 2 H, Ar-CH-O), 3.87 (s, 3H, OMe), 3.86 (s, 3H, OMe), 2.45 (s, 3 H, Me),

2.27 (dd, $J$ = 4.0 and 12.0 Hz, 2 H, C-$CH_{eq}$-C), 1.86 (q, $J$ = 11.5 Hz, 1 H, C-$CH_{ax}$-C), 1.76 (q, $J$ = 12 Hz, 1 H, C-$CH_{ax}$-C). $^{13}$C NMR (125 MHz, $CDCl_3$): δ = 149.2, 145.1, 140.2, 133.5, 131.7, 130.1, 127.8, 118.5, 111.3, 109.6, 78.1, 77.7, 77.0, 56.1, 40.1, 39.9, 21.9. IR (KBr): 3010, 2965, 2924, 2852, 1734, 1630, 1516, 1460, 1361, 1265, 1174, 1070, 1028, 949, 904, 846, 813.7, 668.37 $cm^{-1}$. Anal. Calcd for $C_{26}H_{27}BrO_6S$(547.45): C, 57.04; H, 4.97; S, 5.86. Found: C, 56. 98; H, 4.95; S, 5.75.

**(2*S*,4*S*,6*R*)-2-(4-bromophenyl)-6-(3,4,5-trimethoxyphenyl)-4-tosyloxytetrahydropyran (7b)**

Yellow semisolid; yield: 415.8 mg (72%). $^1$H NMR (500 MHz, $CDCl_3$): δ = 7.81 (d, $J$ = 8 Hz, 2 H, Ar-H), 7.46 (d, $J$ = 8.5 Hz, 2 H, Ar-H), 7.35 (d, $J$ = 8 Hz, 2 H, Ar-H), 7.24-7.22 (m, 2 H, Ar-H), 6.57 (s, 2 H, Ar-H), 4.95 (tt, $J$ = 4.5 and 11.5 Hz, 1 H, H-C-OTs), 4.50-4.47 (m, 2 H, Ar-CH-O), 3.86 (s, 6 H, OMe), 3.82 (s, 3 H, OMe), 2.45 (s, 3 H, Me), 2.32-2.26 (m, 2 H, C-$CH_{eq}$-C), 1.84 (q, $J$ = 12 Hz, 1 H, C-$CH_{ax}$-C), 1.79 (q, $J$ = 11.5 Hz, 1 H, C-$CH_{ax}$-C). $^{13}$C NMR (125 MHz, $CDCl_3$): δ = 153.2, 144.9, 139.8, 137.6, 136.2, 134.2, 131.5, 129.9, 127.6, 121.7, 103.1, 77.8, 77.7, 76.7, 60.8, 56.1, 39.8, 39.7, 21.6. IR (KBr): 2960, 2925, 2843, 1726, 1594, 1461, 1382, 1175, 1126, 1010, 953, 904, 817 $cm^{-1}$. Anal. Calcd for $C_{27}H_{29}BrO_7S$ (577.48): C, 56.16; H, 5.06; S, 5.55. Found: C, 56.20; H, 5.03; S, 5.42.

**(2*R*,4*S*,6*S*)-2,6-bis(4-bromophenyl)-4-tosyloxytetrahydropyran (8b)**

Pale yellow solid; yield: 438 mg (95%); mp 110-112 $^o$C. $^1$H NMR (500 MHz, $CDCl_3$): δ = 7.73 (d, $J$ = 8 Hz, 2 H, Ar-H), 7.38 (m, 4 H, Ar-H), 7.26 (d, $J$ = 8 Hz, 2 H, Ar-H), 7.13 (d, $J$ = 8.5 HZ, 4 H, Ar- H), 4.84 (tt, $J$ = 4.5 and 11.5 Hz, 1 H, H-C-OTs), 4.40 (d, $J$ = 10 Hz, 2 H, Ar-CH-O), 2.37 (s, 3 H, Me), 2.18 (dd, $J$ = 4.5 and 12.5 Hz, 2 H, C-$CH_{eq}$-C), 1.68 (q, $J$ = 11.5 Hz, 2 H, C-$CH_{ax}$-C). $^{13}$C NMR (125 MHz, $CDCl_3$): δ = 145.0, 139.8, 134.2, 131.6, 129.98, 127.6, 121.8, 77.5, 76.8, 39.8, 21.7. IR (KBr): 3100, 2960, 2908, 2843, 1895, 1726, 1595, 1489, 1353, 1172,

1073, 1011, 950, 908, 811, 682, 664 $cm^{-1}$. Anal. Calcd for $C_{24}H_{22}Br_2O_4S$ (566.30): C, 50.90; H, 3.92; S, 5.66. Found: C, 50.82; H, 3.94; S, 5.55.

**(2*S*,4*R*,6*R*)-2-(4-bromophenyl)-6-(4-methoxyphenyl)-4-tosyloxytetrahydropyran (9b)**

Yellow semisolid; yield: 388 mg (75%). $^{1}H$ NMR (500 MHz, $CDCl_3$): δ = 7.74 (d, *J* = 8.5 Hz, 2 H, Ar-H), 7.38 (dd, *J* = 2 and 7 Hz, 2 H, Ar-H), 7.27 (d, *J* = 8.5 Hz, 2 H, Ar-H), 7.21-7.14 (m, 4 H, Ar-H), 6.80 (dd, *J* = 2 and 6.5 Hz, 2 H, Ar-H), 4.84 (tt, *J* = 4.5 and 11 Hz, 1H, H-C-OTs), 4.41 (t, *J* = 11 Hz, 2 H, Ar-CH-O), 3.72 (s, 3 H), 2.38 (s, 3 H, Me), 2.22-2.15 (m, 2 H, C-$CH_{eq}$-C), 1.77 (q, *J* = 12 Hz, 1H, C-CH-$C_{ax}$), 1.73 (q, *J* = 11.5 Hz, 1H, C-$CH_{ax}$-C). $^{13}C$ NMR (125 MHz, $CDCl_3$): δ = 159.3, 144.9, 140.1, 139.8, 137.0, 134.3, 132.9, 131.6, 129.97, 127.6, 127.6, 127.3, 113.9, 78.0, 77.5, 76.8, 55.3, 40.0, 39.8, 21.7. IR (KBr): 2920, 2850, 1631, 1499, 1383, 1299, 1248, 1175, 1073, 904, 813, 730, 721, 669 $cm^{-1}$. Anal. Calcd for $C_{25}H_{25}BrO_5S$(517.43): C, 58.03; H, 4.87; S, 6.20. Found: C, 58.10; H, 4.85; S, 6.10.

**(2*S*,4*R*,6*R*)-2-(4-bromophenyl)-6-(4-chlorophenyl)-4-tosyloxytetrahydropyran (10b)**

Pale yellow solid; yield: 495 mg (95%); mp 110-112 °C. $^{1}H$ NMR (500 MHz, $CDCl_3$): δ = 7.78 (d, *J* = 8 Hz, 2 H, Ar-H), 7.42 (d, *J* = 8.5 Hz, 2 H, Ar-H), 7.31 (d, *J* = 8 Hz, 2 H, Ar-H), 7.28-7.23 (m, 4 H, Ar-H), 7.18 (d, *J* = 8.5 Hz, 2 H, Ar-H), 4.9 (tt, *J* = 4.5 and 11.5 Hz, 1 H, H-C-OTs), 4.46 (t, *J* = 10 Hz, 2 H, Ar-CH-O), 2.40 (s, 3 H, Me), 2.22 (dd, *J* = 3 and 12.5 Hz, 2 H, C-$CH_{eq}$-C), 1.72 (qd, *J* = 2.5 and 11 Hz, 2 H, C-$CH_{ax}$-C). $^{13}C$ NMR (125MHz, $CDCl_3$): δ = 144.9, 139.7, 139.2, 134.1, 133.5, 131.5, 129.9, 128.5, 127.5, 127.1, 121.7, 77.5, 76.7, 39.74, 39.7, 21.6. IR (KBr): 3078, 2965, 2921, 2856, 1908, 1708, 1630, 1591, 1489, 1354, 1171, 1081, 948, 908, 831, 669 $cm^{-1}$. Anal. Calcd for $C_{24}H_{22}BrClO_4S$ (521.85): C, 55.24; H, 4.25; S, 6.14. Found: C, 55.16; H, 4.20; S, 6.22.

**(2*R*,4*R*,6*S*)-2-(4-chlorophenyl)-6-(3-nitrophenyl)-4-tosyloxytetrahydropyran (11b)**

Yellow solid; yield: 468.43 mg (96%); mp 118-120 °C. $^1$H NMR (500 MHz, $CDCl_3$): δ = 8.62 (s, 1 H, Ar-H), 8.4 (dd, *J* = 3 and 8 Hz, 1 H, Ar-H), **,** 8.14 (d, *J* = 7.5 Hz, 1 H, Ar-H), 7.72 (d, *J* = 8.5 Hz, 2 H, Ar-H), 7.67 (t, *J* = 8 Hz, 1 H, Ar-H), 7.26 (d, *J* = 8 Hz, 2 H, Ar-H), 7.229-7.186 (m, 4 H, Ar-H), 4.85 (tt, *J* = 4.5 and 11 Hz, 1 H, H-C-OTs)**,** 4.43 (dd, *J* = 1 and 11.5 Hz, 2 H, Ar-CH-O), 2.36 (s, 3 H, Me), 2.18 (dd, *J* = 4.5 and 12.5 Hz, 2 H, C-$CH_{eq}$-C**),** 1.68 (q, *J* = 11.5 Hz, 2 H, C-$CH_{ax}$-C). $^{13}$C NMR (125 MHz, $CDCl_3$): δ = 148.5, 144.9, 139.0, 137.1, 134.6, 133.7, 133.4, 130.3, 129.8, 128.5, 127.4, 127.1, 124.4, 77.4, 76.7, 39.7, 21.6. IR (KBr): 3069.6, 2960.87, 2926.1, 1703.7, 1596, 1533, 1352, 1171, 1090, 970.9, 908.8, 841, 700, 666 cm$^{-1}$. Anal. Calcd for $C_{24}H_{22}ClNO_6S$ (487.95): C, 59.07; H, 4.54; N, 2.87; S, 6.57. Found: C, 58.93; H, 4.58; N, 2.82; S, 6.40.

**(2*R*,4*R*,6*S*)-2-phenyl-6-(3,4,5-trimethoxyphenyl)-4-tosyloxytetrahydropyran (12b)**

Dark yellow solid; yield: 413.8 mg (83%); mp 116-118 °C. $^1$H NMR (500 MHz, $CDCl_3$): δ = 7.85 (d, *J* = 8 Hz, 2 H, Ar-H), 7.38 (m, 7 H, Ar-H), 6.6 (s, 2 H, Ar-H), 4.99 (tt, *J* = 4.5 and 11 Hz, 1 H, H-C-OTs), 4.52 (ddd, *J* = 2, 11.5 and 25.5 Hz, 2 H, Ar-CH-O), 3.9 (s, 6 H, OMe), 3.84 (s, 3 H, OMe), 2.47 (s, 3 H, Me), 2.34-2.29 (m, 2 H, C-$CH_{eq}$-C), 1.93-1.84 (m, 2 H, C-$CH_{ax}$-C). $^{13}$C NMR (125 MHz, $CDCl_3$): δ = 152.3, 143.8, 143.1, 139.8, 136.5, 135.5, 128.9, 127.5, 126.9, 126.6, 124.9, 102.2, 77.1, 76.8, 75.7, 59.8, 55.1, 39.0, 38.9, 20.6. IR (KBr): 2962, 2926, 2852, 1634, 1595, 1456, 1417, 1261, 1178, 1096, 1023, 803, 873, 708 cm$^{-1}$. Anal. Calcd for $C_{27}H_{30}O_7S$ (498.6): C, 65.05; H, 6.06; S, 6.43. Found: C, 65.10; H, 6.08; S, 6.38.

**(2*R*,4*S*,6*S*)-2-(4-chlorophenyl)-6-(4-methoxyphenyl)-4-tosyloxytetrahydropyran (13b)**

Yellow semisolid; yield: 416 mg (88%). $^1$H NMR (500 MHz, $CDCl_3$): δ = 7.74 (d, *J* = 8.5 Hz, 2 H, Ar-H), 7.28-7.19 (m, 8 H, Ar-H), 6.8 (d, *J* = 9 Hz, 2 H, Ar-H), 4.85 (tt, *J* = 4.5 and 11.5 Hz, 1

H, H-C-OTs), 4.45-4.39 (ddd, *J* = 1.5, 11.5 and 16.5 Hz, 2 H, Ar-CH-O), 3.72 (s, 3 H, OMe), 2.38 (s, 3 H, Me), 2.22-2.15 (m, 2 H, C- $CH_{eq}$-C), 1.76 (q, *J* = 11.5 Hz, 2 H, C-$CH_{ax}$-C), 1.7 (q, *J* = 11.5 Hz, 2 H, C-$CH_{ax}$-C). $^{13}$C NMR (125 MHz, $CDCl_3$): δ = 159.3, 144.8, 141.0, 133.2, 129.9, 128.5, 128.4, 127.9, 127.6, 127.3, 126.0, 113.8, 78.38, 76.8, 55.3, 40.1, 39.9, 39.6, 21.7. IR (KBr): 3010, 2956, 2921, 2847, 1721, 1633, 1508, 1456, 1386, 1252, 1178, 1082, 1026, 904, 813 $cm^{-1}$. Anal. Calcd for $C_{25}H_{25}ClO_5S$ (472.9): C, 63.48; H, 5.33; S, 6.78. Found: C, 63.45; H, 5.36; S, 6.69.

**(2*S*,4*R*,6*R*)-2-(4-bromophenyl)-6-phenyl-4-tosyloxytetrahydropyran (14b)**

Pale yellow solid; yield: 463 mg (95%); mp 104-106 °C. $^1$H NMR (500 MHz, $CDCl_3$): δ = 7.84-7.22 (m, 2 H, Ar-H), 7.47 (dd, *J* = 2 and 8.5 Hz, 2 H, Ar-H), 7.39-7.22 (m, 9 H, Ar-H), 4.95 (m, 1 H, H-C-OTs), 4.56-4.48 (m, 2 H, Ar-CH-O), 2.45 (s, 3 H, Me), 2.30-2.27 (m, 2 H, C-CH-$C_{eq}$), 1.89-1.74 (m, 2 H, C-CH-$C_{ax}$). $^{13}$C NMR (125MHz, $CDCl_3$): δ = 143.9, 139.7, 139.0, 133.2, 130.5, 128.9, 127.4, 126.9, 126.6, 124.8, 120.7, 76.8, 76.51, 75.79, 38.9, 38.8, 20.6. IR (KBr): 2921.7, 1630, 1486, 1352, 1182, 1065, 1008, 900, 817, 756 $cm^{-1}$. Anal. Calcd for $C_{24}H_{23}BrO_4S$ (487.4): C, 59.14; H, 4.76; S, 6.58. Found: C, 59.12; H, 4.79; S, 6.49.

**(2*R*,4*S*,6*S*)-2-(4-chlorophenyl)-6-*p*-tolyl-4-tosyloxytetrahydropyran (15b)**

Yellow solid; yield: 425 mg (93%); mp 104-106 °C. $^1$H NMR (500MHz, $CDCl_3$): δ = 7.81 (d, *J* = 8 Hz, 2 H, Ar-H), 7.34 (d, *J* = 8.5 Hz, 2 H, Ar-H), 7.30- 7.22 (m, 6 H, Ar-H), 7.146 (d, *J* = 7.5 Hz, 2 H, Ar-H), 4.92 (tt, *J* = 4.5 and 11 Hz, 1 H, H-C-OTs), 4.49 (td, *J* = 2 and 11 Hz, 2 H, Ar-CH-O), 2.45 (s, 3 H), 2.33 (s, 3 H, Me), 2.29-2.26 (m, 2 H, C-$CH_{eq}$-C), 1.84-1.76 (m, 2 H, C-$CH_{ax}$-C). $^{13}$C NMR (125MHz, $CDCl_3$): δ = 144.9, 139.6, 137.8, 134.3, 133.5, 129.96, 129.2, 128.6, 127.6, 127.3, 125.9, 78.0, 77.54, 40.4, 39.8, 21.7, 21.2. IR (KBr): 3047, 2982, 2916, 2843,

2373, 1639, 1369, 1265, 1169, 1117, 738 $cm^{-1}$. Anal. Calcd for $C_{25}H_{25}ClO_4S$ (456.98): C, 65.71; H, 5.51; S, 7.02. Found: C, 65.76; H, 5.49; S, 7.15.

**(2*S*,4*R*,6*R*)-2-(furan-2-yl)-6-phenyl-4-tosyloxytetrahydropyran (16b)**

Yellow solid; yield: 330 mg (83%); mp 123-125 $^oC$. $^1H$ NMR (500MHz, $CDCl_3$): δ = 7.82 (d, *J* = 7.5 Hz, 2 H, Ar-H), 7. 41 (d, *J* = 7.5 Hz, 2 H, Ar-H), 7.31 (m, 1 H, Ar-H), 7.21 (m, 5 H, Ar-H), 6.43-6.25 (m, 2 H, Ar-H), 5.15 (t, *J* = 12Hz 1H,), 4.95 (tt, *J* = 4.5 and 11 Hz, 1 H, H-C-OTs), 4.53 (td, *J* = 2 and 11.5 Hz, 1 H, Ar-CH-O), 2.45 (s, 3 H), 2.25-2.21 (m, 2 H, C-$CH_{eq}$-C), 1.90-1.86 (m, 2 H, C-$CH_{ax}$-C). $^{13}C$ NMR (125MHz, $CDCl_3$): δ = 153.2, 144.5, 142.2, 139.8, 138.4, 132.7, 130.9, 128.6, 128.5, 127.0, 112.4, 111.6, 78.0, 75.2, 40.7, 39.9, 22.5. IR (KBr): 3021, 2986, 2355, 1696, 1432 $cm^{-1}$. Anal. Calcd for $C_{22}H_{22}O_5S$ (398.47): C, 66.31; H, 5.56; S, 8.05. Found: C, 66.24; H, 5.52; S, 8.20.

**(2*S*,4*R*,6*R*)-2-phenyl-6-(1*H*-pyrrol-2-yl)-4-tosyloxytetrahydropyran (17b)**

Yellow semi solid; yield: 341 mg (86%); $^1H$ NMR (500MHz, $CDCl_3$): δ = 8.21 (s, br, $D_2O$ exchangeable, 1H, NH), 7.81 (d, *J* = 7.5 Hz, 2 H, Ar-H), 7. 44 (d, *J* = 7.5 Hz, 2 H, Ar-H), 7.19 (m, 5 H, Ar-H), 6.43 (m, 1 H, Ar-H), 5.95-5.7 (m, 2H, Ar-H), 4.93 (tt, *J* = 4.5 and 11.5 Hz, 1 H, H-C-OTs), 4.87 (td, *J* = 2 and 11.5 Hz, 2 H, Ar-CH-O), 2.41 (s, 3 H), 2.25-2.21 (m, 2 H, C-$CH_{eq}$-C), 1.90-1.86 (m, 2 H, C-$CH_{ax}$-C). $^{13}C$ NMR (125MHz, $CDCl_3$): δ = 144.3, 140.3, 139.1, 133.6, 130.8, 128.9, 128.2, 127.2, 118.5, 108.6, 107.4, 77.6, 72.1, 59.8, 41.4, 40.2, 21.3. IR (KBr): 3451, 3069.6, 2960.87, 1703.7, 1596, 1533, 1352 $cm^{-1}$. Anal. Calcd for $C_{22}H_{23}NO_4S$ (397.13): C, 66.48; H, 5.83; N, 3.52; S, 8.07. Found: C, 66.40; H, 5.81; N, 3.60; S, 8.20.

**(2*R*,4*S*,6*S*)-2-methyl-6-phenyl-4-tosyloxytetrahydropyran (18b):**

Yellow solid; yield: 307 mg (89%); mp 92-94 $^oC$. $^1H$ NMR (500 MHz, $CDCl_3$): δ = 7.88 (d, *J* = 8 Hz, 2 H, Ar-H), 7.34-7.31 (d, *J* = 8 Hz, 2 H, Ar-H), 7.22 (m, 5H), 5.03 (tt, *J* = 4.5 and 11.5 Hz, 1

H, H-C-OTs), 4.59 (t, *J* = 11.5 Hz, 1 H, Ar-CH-O), 3.92 (m, 1 H), 2.48 (s, 3 H, Me), 2.33-2.37 (dd, *J* = 4.5 and 11.5 Hz, 2 H, C-$CH_{eq}$-C), 1.88 (q, *J* = 7.5 Hz, 2 H, C-$CH_{ax}$-C). 1.56 (m, 3H). $^{13}$CNMR (125 MHz, $CDCl_3$): δ = 144.7, 140.9, 134.2, 129.8, 128.3, 127.7, 127.5, 125. 82, 77.4, 68.21, 61.2, 39.9, 24.3, 21.6. IR (KBr): 3056, 2923, 2852, 1717, 1629, 1454, 1379 cm$^{-1}$. Anal. Calcd for $C_{19}H_{22}O_4S$ (346.12): C, 65.87; H, 6.40; S, 9.26. Found: C, 65.85; H, 6.36; S, 9.20.

**(2*R*,4*s*,6*S*)-2,6-bis(4-chlorophenyl)tetrahydropyran-4-ol (1c)**

Yellow solid; yield: 297 mg (92%); mp 110-112 °C. $^1$H NMR (500 MHz, $CDCl_3$): δ = 7.29-7.24 (m, 8 H, Ar-H), 4.47 (d, *J* = 11.5 Hz, 2 H, Ar-CH-O), 4.06 (tt, *J* = 4.5 and 11 Hz, 1 H, H-C-OH), 2.19 (dd, *J* = 4 and 12 Hz, 2 H, C-$CH_{eq}$-C), 1.48 (q, *J* = 11.5 Hz, 2 H, C-$CH_{ax}$-C). $^{13}$C NMR (125 MHz, CDCl3): δ = 139.2, 132.3, 127.5, 126.2, 67.4, 41.9. IR (KBr): 3447.1, 2960.87, 2886, 1652, 1543, 1088, 804 cm$^{-1}$. GC-MS: *m*/*z* (%) = 323 (65, [M$^+$]), 138(100).

**(2*R*,4*s*,6*S*)-2,6-diphenyltetrahydropyran-4-ol (2c)**

Yellow solid; yield: 241.3 mg (95%); mp 102-103 °C. $^1$H NMR (500 MHz, $CDCl_3$): δ = 7.42 (d, *J* = 7 Hz, 4 H, Ar-H), 7.35 (t, *J* = 7.5 Hz, 4 H, Ar-H), 7.28 (m, 2 H, Ar-H), 4.57 (d, *J* = 11.5 Hz, 2 H, Ar-CH-O), 4.16 (tt, *J* = 4.5 and 11 Hz, 1 H, H-C-OH), 2.29 (dd, *J* = 2 and 10 Hz, 2 H, C-$CH_{eq}$-C), 2.23 (s, br, $D_2O$ exchangeable, 1 H, OH), 1.60 (q, *J* = 11.5 Hz, 2 H, C-$CH_{ax}$-C). $^{13}$C NMR (125 MHz, $CDCl_3$): δ = 141.8, 128.2, 127.4, 125.7, 77.8, 68.6, 42.96. IR (KBr): 3434, 3010, 2922, 2843, 1734, 1626, 1456, 1386, 1256, 1069, 808.8 cm$^{-1}$. GC-MS: *m*/*z* (%) = 254 (38, [M$^+$]), 104(100).

**(2*R*,4*s*,6*S*)-2,6-bis(4-bromophenyl)tetrahydropyran-4-ol (3c)**

Yellow solid; yield: 385 mg (94%); mp 122-123 °C. $^1$HNMR (500 MHz, $CDCl_3$): δ = 7.19-7.41 (m, 8 H, Ar-H), 4.51-4.43 (m, 2 H, Ar-CH-O), 4.07 (tt, *J* = 4.5 and 11.5 Hz, 1 H, H-C-OH), 2.28 (s, br, $D_2O$ exchangeable, 1 H, OH), 2.21 (m, 2 H, C-$CH_{eq}$-C), 1.53 (m, 2 H, C-$CH_{ax}$-C). $^{13}$C

NMR (125 MHz, $CDCl_3$): δ = 131.4, 128.3, 127.5, 125.8, 77.8, 68.6, 43.0, 42.8. IR (KBr): 3433.8, 2965.2, 2921.7, 2852.1, 1634.8, 1452.1, 1382.6, 1265.2, 1156.5, 1065.2, 900.0, 760.87, 700 $cm^{-1}$. GC-MS: *m/z* (%) = 410 (48, [$M^+$]), 104 (100).

**1-Phenylbut-3-en-1-ol (1a)**[1]

Color less oil; yield: 782.5 mg (86%). $^1$H NMR (500 MHz, $CDCl_3$): δ = 7. 3 (d, *J* = 4 $H_{Z,,}$ 2 H, Ar-H), 7.2 (d, *J* = 4 $H_{Z,,}$ 2 H, Ar-H), 5.7 (m, 1 H, C=C-H), 5.1 (m, 2 H, C=C-$H_2$), 4.6 (t, *J* = 5 Hz, 1 H, Ar-CH-OH), 3.2 (s, br, $D_2O$ exchangeable, 1 H, OH), 2.4 (m, 2 H, =C-CH-C). $^{13}$C NMR (125 MHz, $CDCl_3$) δ = 143.8, 134.4, 128.4, 127.6, 125.8, 125.8, 118.5, 73.3, 43.9. IR (KBr): 3450, 3070, 2935, 2917, 1639, 1491, 1417, 1260, 1099, 1012, 917, 835, 739 $cm^{-1}$. GC-MS: *m/z* (%) = 148 (53, [$M^+$]), 107(100).

**1-(4-chlorophenyl)but-3-en-1-ol (2a)**[2]

Pale yellow oil; yield: 651 mg (88%). $^1$H NMR (500 MHz, $CDCl_3$): δ = 7.19 (m, 1 H, Ar-H), 5.7 (m, 1 H, C=C-H), 5.0 (m, 1 H, C=C-$H_2$), 4.6 (t, *J* = 5 Hz, 1 H, Ar-CH-OH), 2.4 (m, 2 H, =C-CH-C), 2.3 (s, br, $D_2O$ exchangeable, 1 H, OH). $^{13}$C NMR (125 MHz, $CDCl_3$): δ = 143.8, 134.5, 128.4, 127.6, 125.8, 125.8, 118.5, 73.3, 43.9. IR (KBr): 3425, 3072, 2923, 2852, 1638, 1443, 1262, 1094, 1030, 804, 701 $cm^{-1}$. GC-MS: *m/z* (%) = 182 (74, [$M^+$]), 141(100).

**1-(4-methoxyphenyl)but-3-en-1-ol (3a)**[3]

Pale yellow oil. Yield: 756 mg (85%). $^1$H NMR (500 MHz, $CDCl_3$): δ = 7.28 (d, *J* = 9 $H_{Z,}$ 2 H, Ar-H), 6.88 (d, *J* = 8.5 $H_{Z,}$ 2 H, Ar-H), 5.82 (m, 2 H, C=C-H), 4.68 (t, *J* = 6.5 Hz, 1 H, Ar-CH-OH), 2.49 (t, *J* = 7 Hz, 2 H, =C-CH-C), 2.1 (s, br, $D_2O$ exchangeable, 1H, OH). $^{13}$C NMR (125 MHz, $CDCl_3$): δ = 158.2, 135.4, 133.9, 126.3, 117.0, 113.2, 72.0, 54.0, 42.0. IR (KBr): 3421.5,

---

[1] Lee, K.; Kim, H.; Hong, J. *Org. Lett.* **2009**, *11*, 5202-5205.
[2] Cui, Y. M.; Huang, Q. Q.; Xu, J.; Chen, L. L.; Li, J. Y.; Ye, Q. Z.; Li, J.; Nan, F. J. *Bioorg. Med. Chem. Lett.* **2005**, *15*, 4130-4135.
[3] Wang, L.; Li, P.; Menche, D. *Angew. Chem. Int. Ed.* **2010**, *49*, 9270-9273

3073.7, 2998.0, 2925.9, 1617, 1508, 1447, 1299, 1247, 1178, 1039, 926, 830, 739 $cm^{-1}$. GC-MS: *m*/*z* (%) = 178 (38, [$M^+$]), 137(100).

**1-(4-bromophenyl)but-3-en-1-ol (4a)**[4]

Pale yellow oil; yield: 995 mg (88%). $^1$H NMR (500 MHz, $CDCl_3$): δ = 7.4 (d, *J* = 7 $H_Z$, 2 H, Ar-H), 7.1 (d, *J* = 10.5 $H_Z$, 2 H, Ar-H), 5.7 (m,1 H), 5.1(m, 2 H), 4.6 (td, *J* = 6.5, 17 Hz, 1 H), 2.49 (m, 2 H), 2.49 (s, br, $D_2O$ exchangeable, 1H, OH). $^{13}$C NMR (125 MHz, $CDCl_3$) δ = 143.8, 134.4, 128.4, 127.6, 125.8, 125.8, 118.5, 73.3, 43.9. IR (KBr): 3406.2, 3077.4, 2978.2, 2928.6, 1640.3, 1591.3, 1487.7, 1405, 1068.6, 1008.6, 910, 825.7, 735.9, 649.27, 535.1 $cm^{-1}$. GC-MS: *m*/*z* (%) = 226 (48, [$M^+$]), 78(100).

**1-(3,4,5-trimethoxyphenyl)but-3-en-1-ol (5a)**[5]

Pale yellow oil; yield: 1046 mg (82%). $^1$H NMR (500 MHz, $CDCl_3$) δ = 6.57 (s, 2 H, Ar-H), 5.8 (m, 1 H), 5.16 (m, 2 H), 4.64 (m, 1 H), 2.49 (m, 2 H), 2.2 (s, broad-OH, $D_2O$ exchangeable, 1 H). $^{13}$C NMR (125 MHz, $CDCl_3$): δ = 153.0, 140.0, 136.9, 134.6, 118.0, 102.6, 73.5, 60.7, 56.0, 43.9. IR (KBr): 3442, 3100, 2934.8, 2847.83, 1637.1, 1593.9, 1460.3, 1421.7, 1330.4, 1233.9, 1126.2, 1060.9, 1004.35, 913.04, 834.8, 669.57 $cm^{-1}$. GC-MS: *m*/*z* (%) = 238 (64, [$M^+$]), 197(100).

---

[4] Mosher, M. D.; Emmerich, L. G.; Frost, K. S.; Anderson, B. *J. Heterocycl. Chem.***2006**, *43*, 535-539.
[5] Mitra, S.; Gurrala, S. R.; Coleman, R. S. *J. Org. Chem.* **2007**, *72,* 8724-8736.

## 6. [1]H data for 4-tosyloxy and 4-hydroxytetrahydropyran:

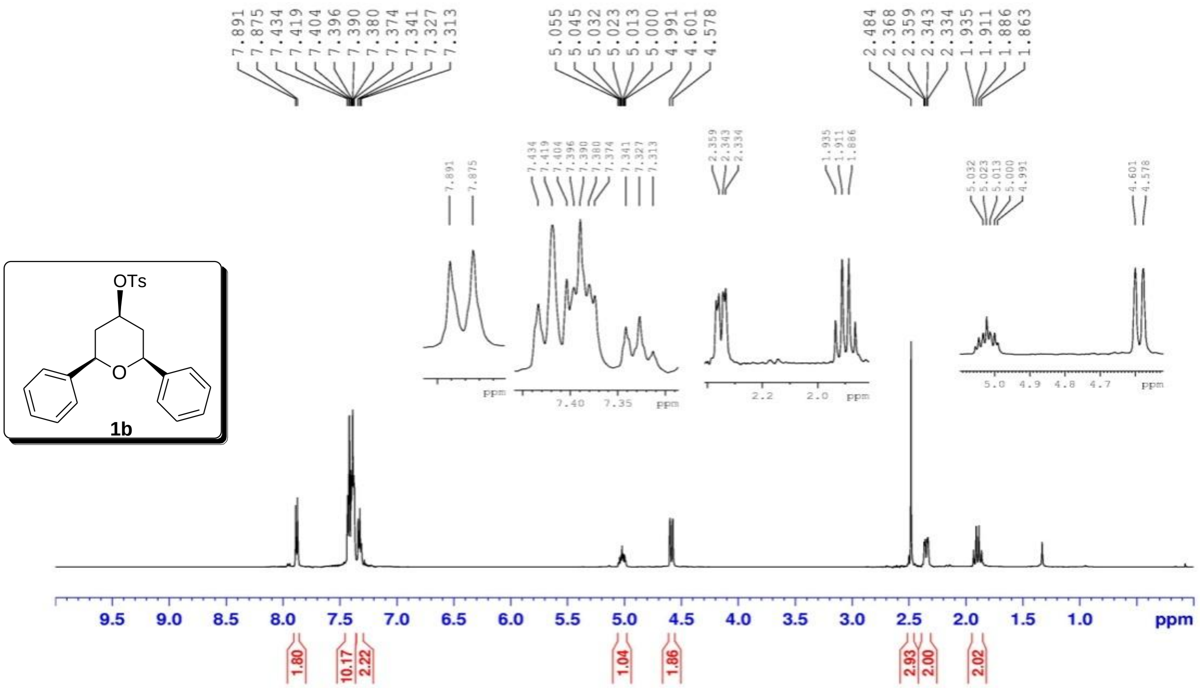


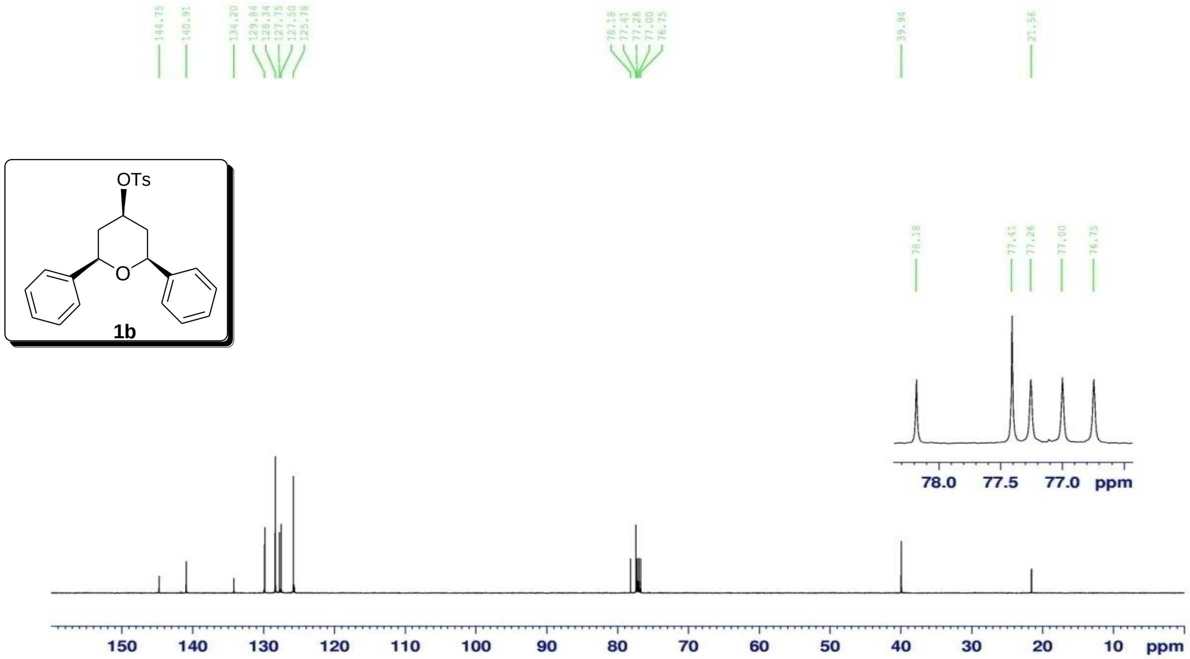

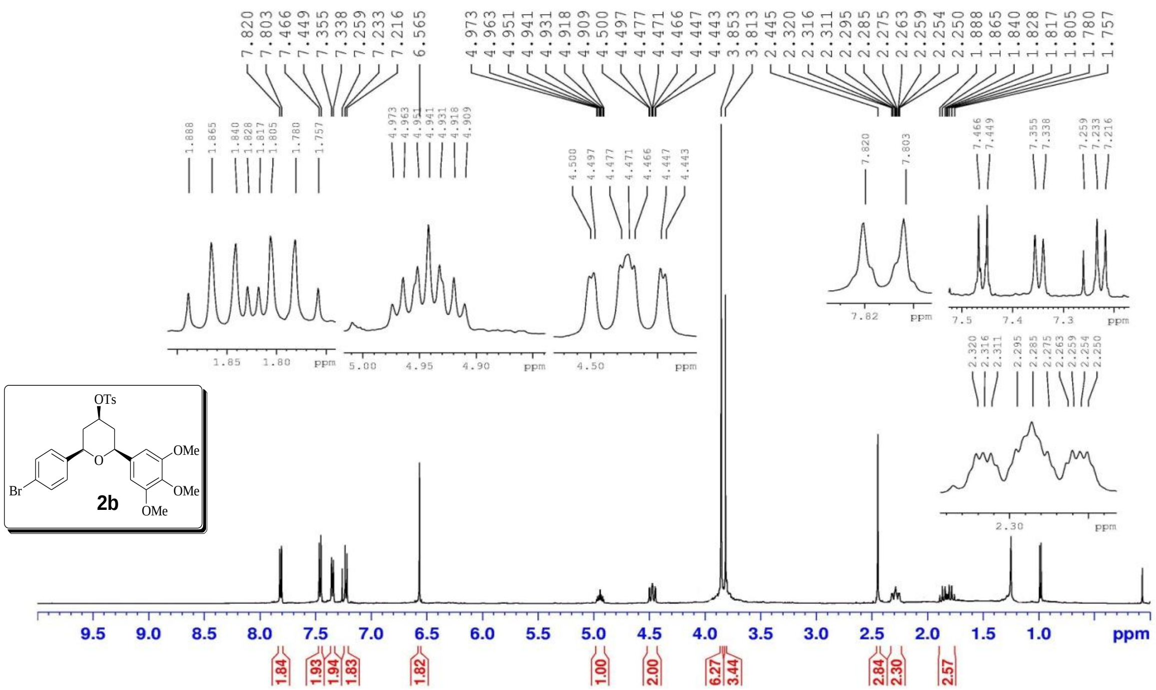
OTs
OMe
Br
O
OMe
2b
OMe
ppm
1.84
1.93
1.94
1.83
1.82
1.00
2.00
6.27
3.44
2.84
2.30
2.57

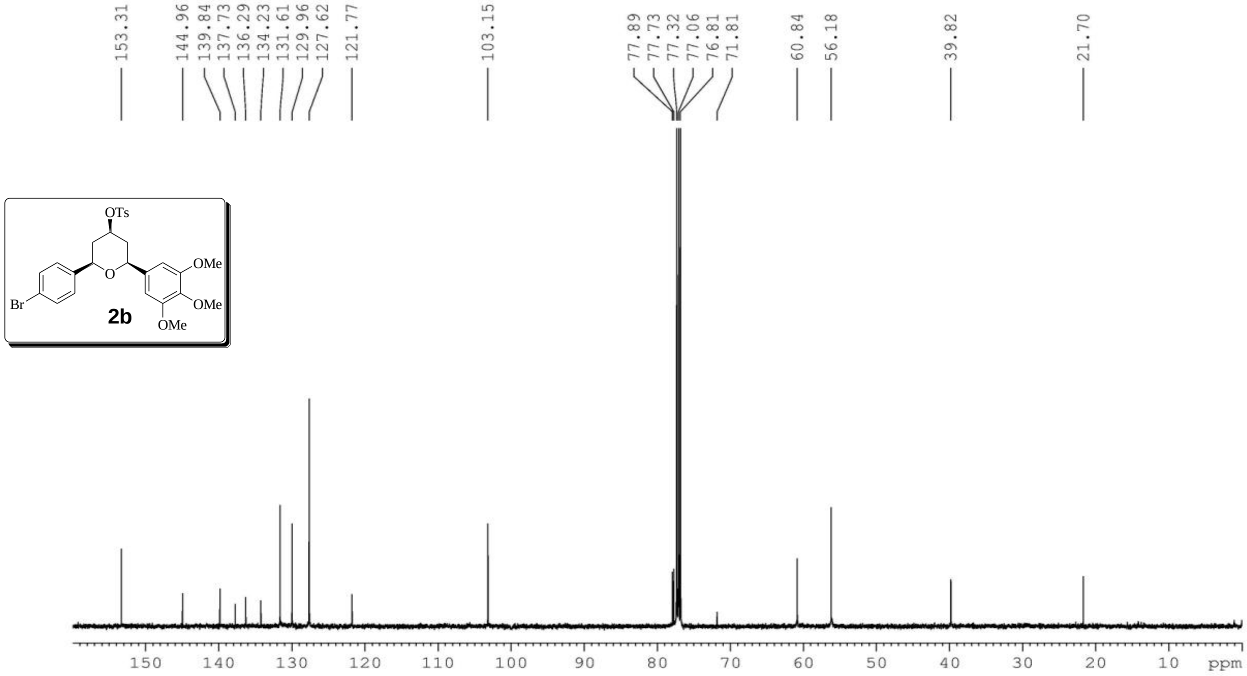
153.31
144.96
139.84
137.73
136.29
134.23
131.61
129.96
127.62
121.77
103.15
77.89
77.73
77.32
77.06
76.81
71.81
60.84
56.18
39.82
21.70
OTs
OMe
Br
O
OMe
2b
OMe
ppm

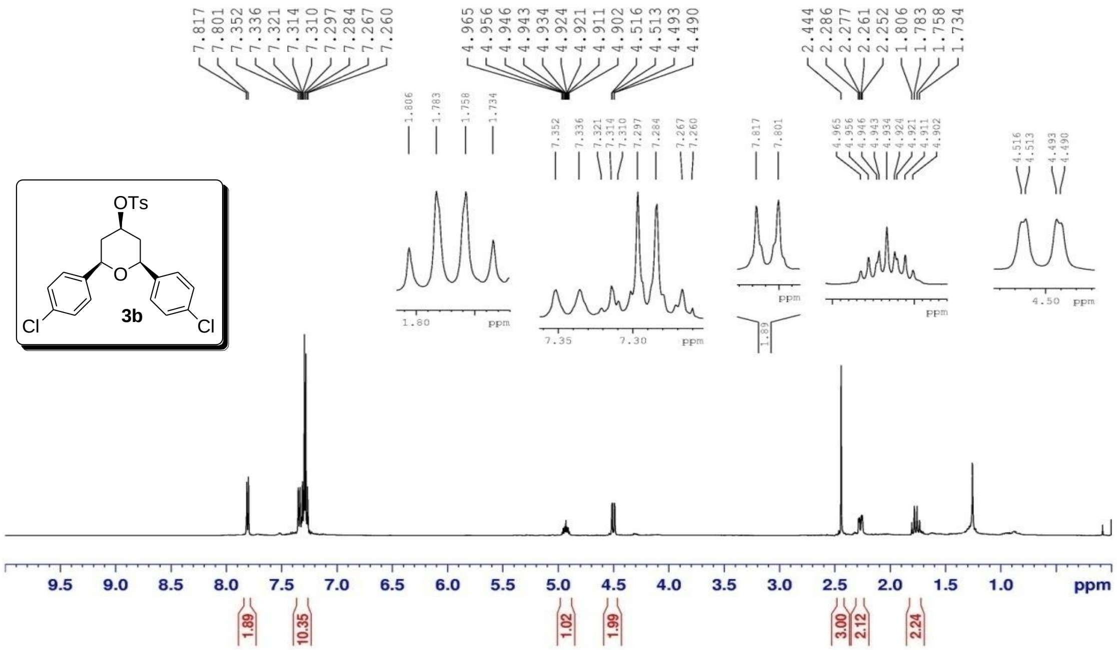
OTs
O
Cl
Cl
3b
7.817
7.801
7.352
7.336
7.321
7.314
7.310
7.297
7.284
7.267
7.260
4.965
4.956
4.946
4.943
4.934
4.924
4.921
4.911
4.902
4.516
4.513
4.493
4.490
2.444
2.286
2.277
2.261
2.252
1.806
1.783
1.758
1.734
9.5 9.0 8.5 8.0 7.5 7.0 6.5 6.0 5.5 5.0 4.5 4.0 3.5 3.0 2.5 2.0 1.5 1.0 ppm
1.89
10.35
1.02
1.99
3.00
2.12
2.24

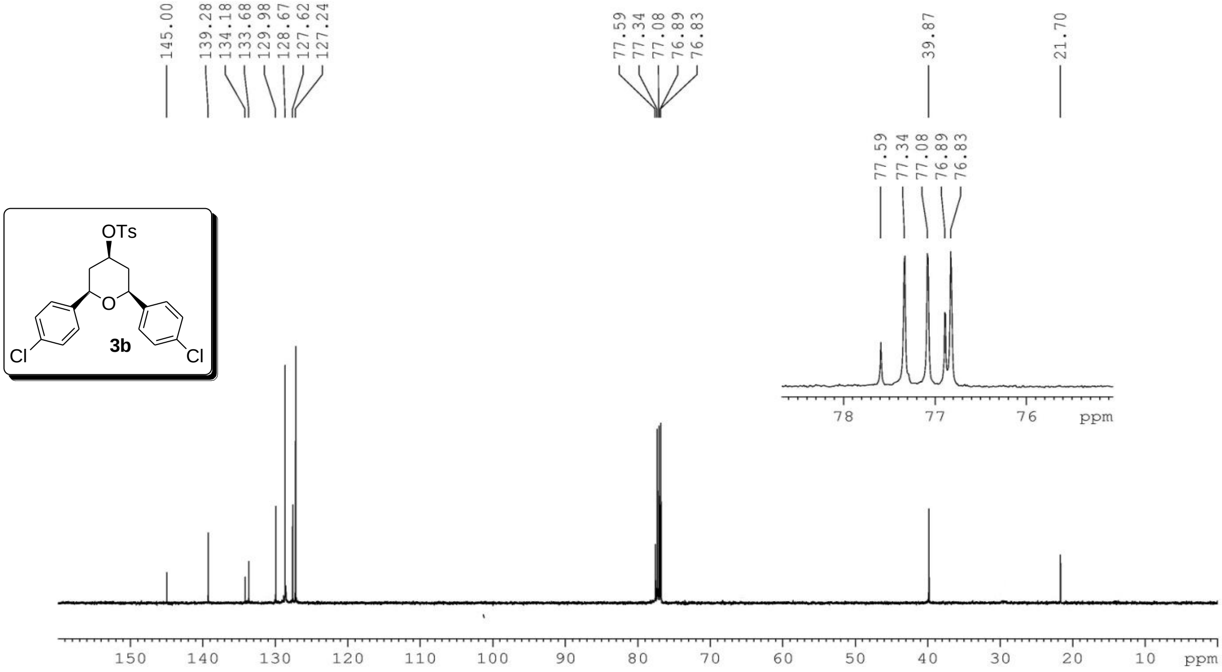
145.00
139.28
134.18
133.68
129.98
128.67
127.62
127.24
77.59
77.34
77.08
76.89
76.83
39.87
21.70
OTs
O
Cl
Cl
3b
78 77 76 ppm
150 140 130 120 110 100 90 80 70 60 50 40 30 20 10 ppm

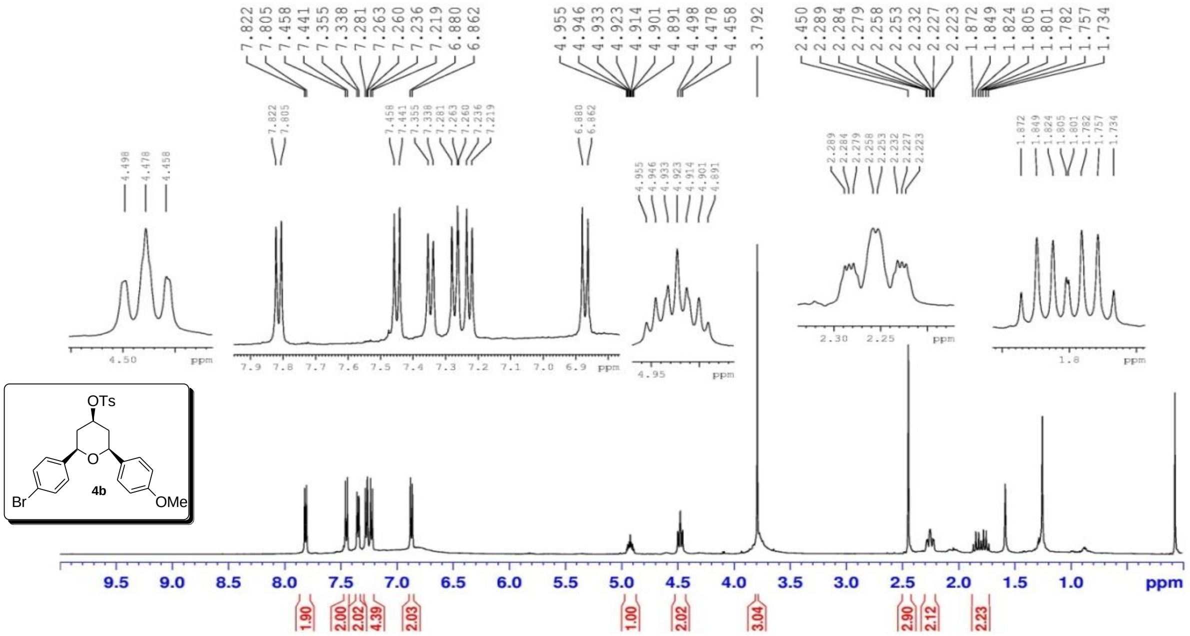
7.822
7.805
7.458
7.441
7.355
7.338
7.281
7.263
7.260
7.236
7.219
6.880
6.862
4.955
4.946
4.933
4.923
4.914
4.901
4.891
4.498
4.478
4.458
3.792
2.450
2.289
2.284
2.279
2.258
2.253
2.232
2.227
2.223
1.872
1.849
1.824
1.805
1.801
1.782
1.757
1.734
OTs
O
Br
4b
OMe
ppm
1.90
2.00
2.02
4.39
2.03
1.00
2.02
3.04
2.90
2.12
2.23

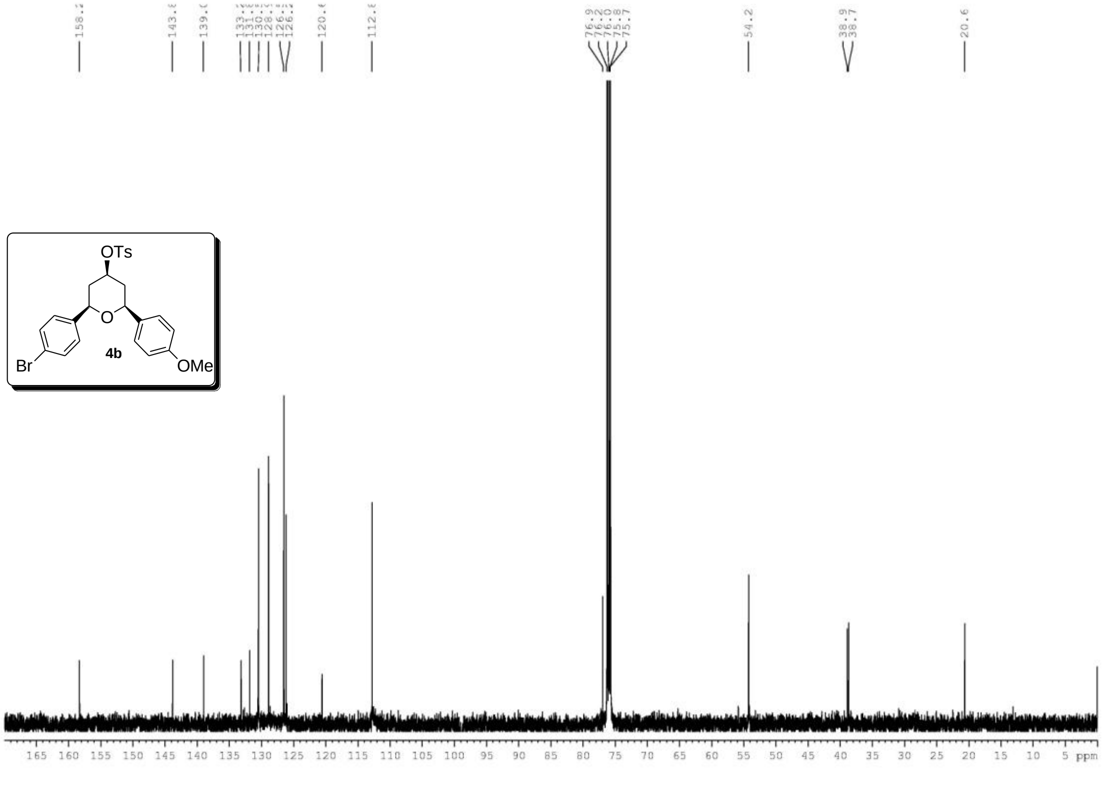
54.2
38.9
38.7
20.6
OTs
O
Br
4b
OMe
ppm

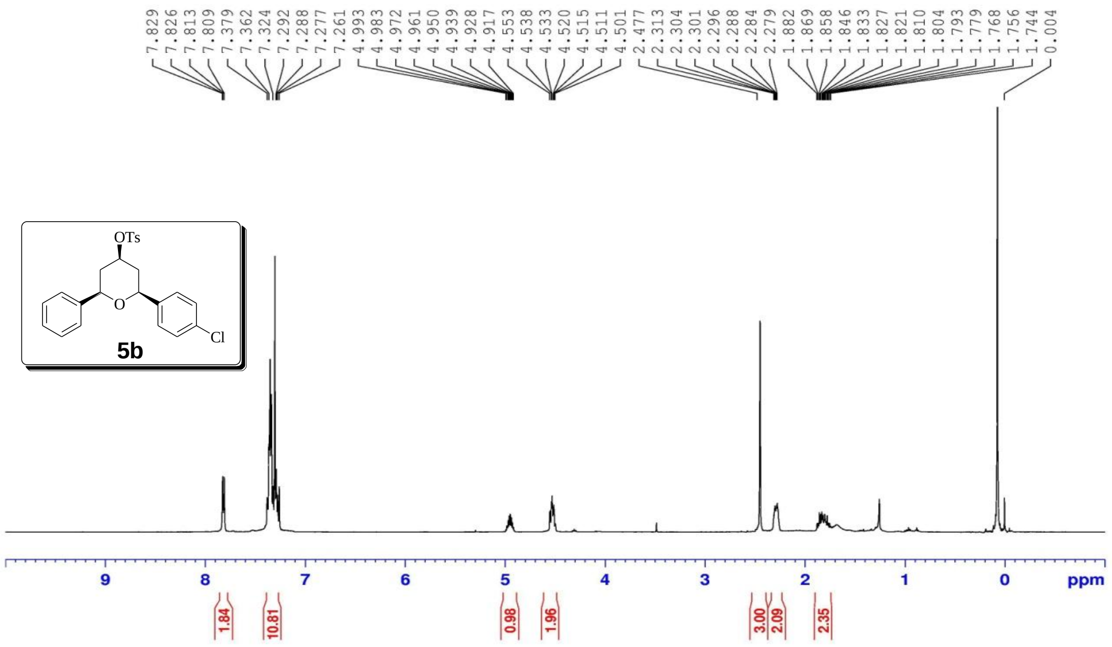
7.829 7.826 7.813 7.809 7.379 7.362 7.324 7.292 7.288 7.277 7.261 4.993 4.983 4.972 4.961 4.950 4.939 4.928 4.917 4.553 4.538 4.533 4.520 4.515 4.511 4.501 2.477 2.313 2.304 2.301 2.296 2.288 2.284 2.279 1.882 1.869 1.858 1.846 1.833 1.827 1.821 1.810 1.804 1.793 1.779 1.768 1.756 1.744 0.004
OTs
O
Cl
5b
9 8 7 6 5 4 3 2 1 0 ppm
1.84 10.81 0.98 1.96 3.00 2.09 2.35

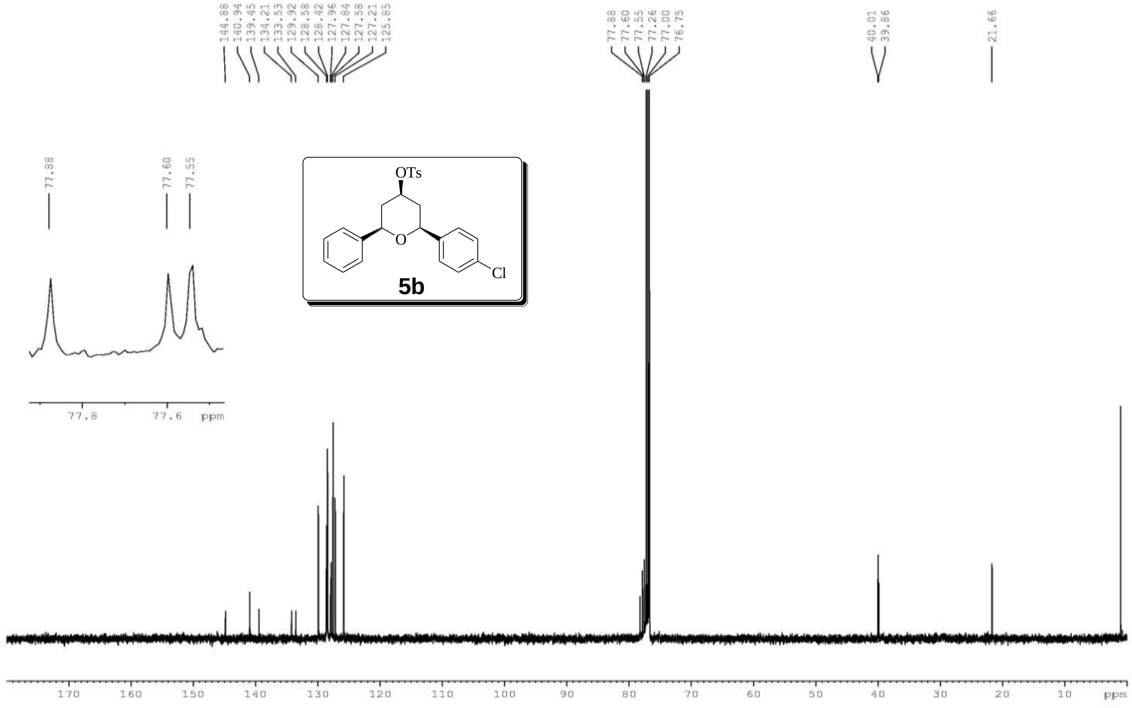
144.88 140.94 139.45 134.21 133.53 129.92 128.58 128.42 127.96 127.84 127.58 127.21 125.85
77.88 77.60 77.55 77.26 77.00 76.75
40.01 39.86
21.66
77.88 77.60 77.55
77.8 77.6 ppm
OTs
O
Cl
5b
170 160 150 140 130 120 110 100 90 80 70 60 50 40 30 20 10 ppm

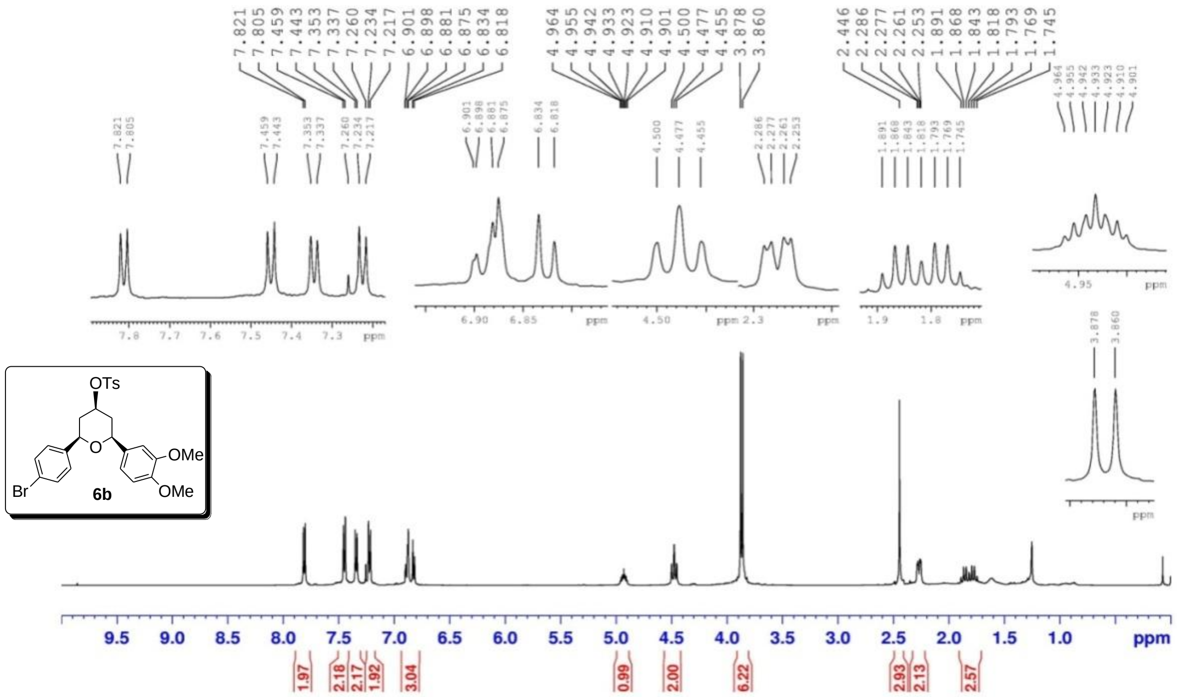
7.821
7.805
7.459
7.443
7.353
7.337
7.260
7.234
7.217
6.901
6.898
6.881
6.875
6.834
6.818
4.964
4.955
4.942
4.933
4.923
4.910
4.901
4.500
4.477
4.455
3.878
3.860
2.446
2.286
2.277
2.261
2.253
1.891
1.868
1.843
1.818
1.793
1.769
1.745
OTs
O
OMe
OMe
Br
6b
9.5 9.0 8.5 8.0 7.5 7.0 6.5 6.0 5.5 5.0 4.5 4.0 3.5 3.0 2.5 2.0 1.5 1.0 ppm
1.97
2.18
2.17
1.92
3.04
0.99
2.00
6.22
2.93
2.13
2.57

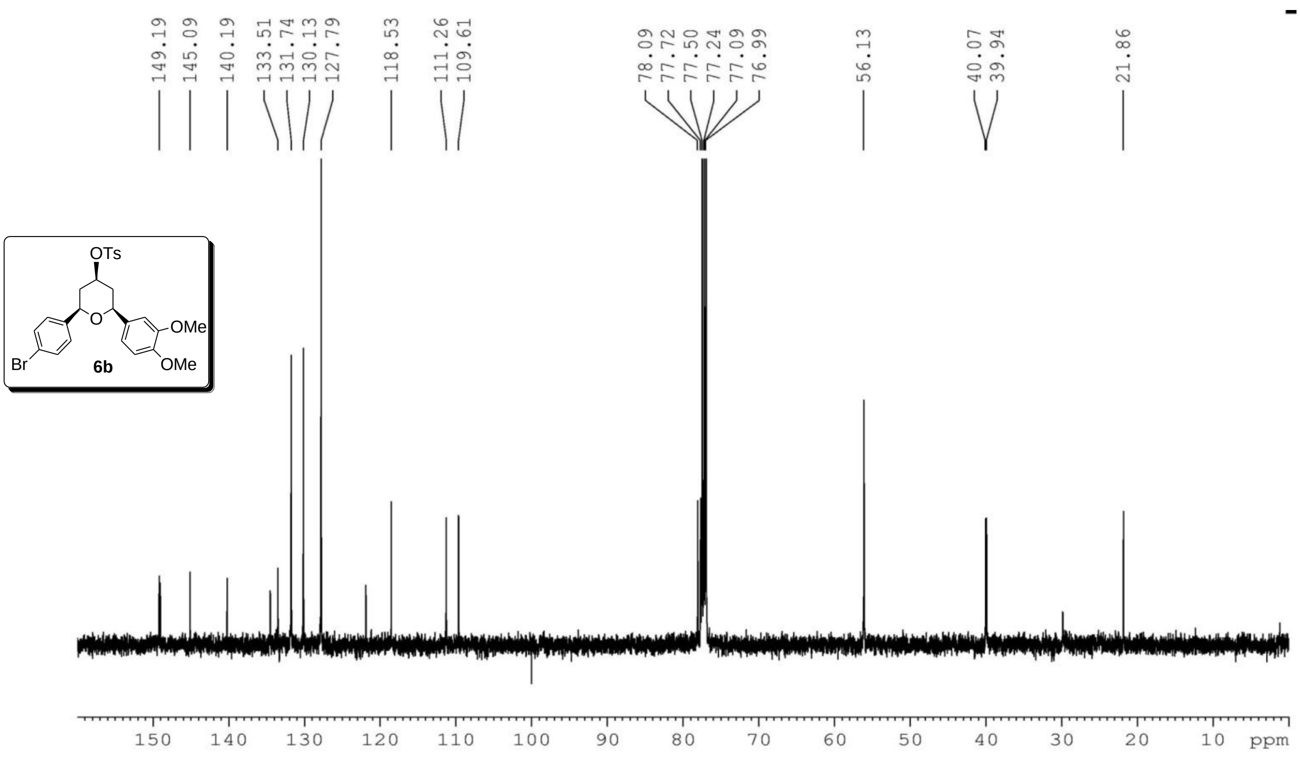
149.19
145.09
140.19
133.51
131.74
130.13
127.79
118.53
111.26
109.61
78.09
77.72
77.50
77.24
77.09
76.99
56.13
40.07
39.94
21.86
OTs
O
OMe
OMe
Br
6b
150 140 130 120 110 100 90 80 70 60 50 40 30 20 10 ppm

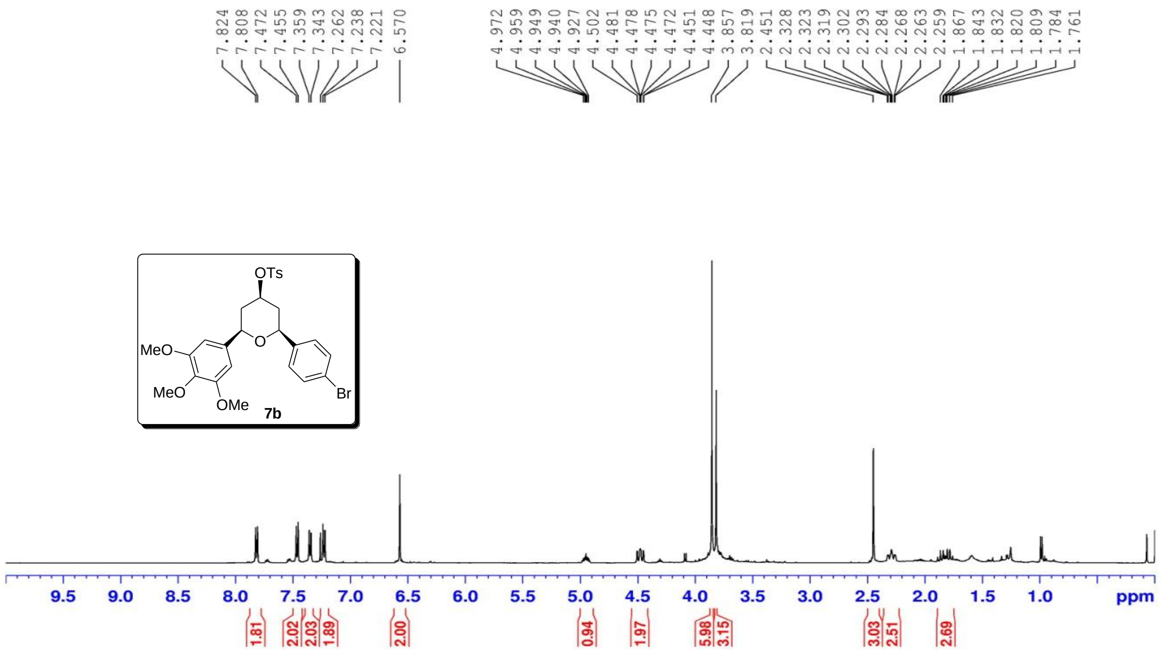
7.824 7.808 7.472 7.455 7.359 7.343 7.262 7.238 7.221 6.570
4.972 4.959 4.949 4.940 4.927 4.502 4.481 4.478 4.475 4.472 4.451 4.448 3.857 3.819 2.451 2.328 2.323 2.319 2.302 2.293 2.284 2.268 2.263 2.259 1.867 1.843 1.832 1.820 1.809 1.784 1.761
OTs
MeO
MeO
OMe
O
Br
7b
9.5 9.0 8.5 8.0 7.5 7.0 6.5 6.0 5.5 5.0 4.5 4.0 3.5 3.0 2.5 2.0 1.5 1.0 ppm
1.81 2.02 2.03 1.89 2.00 0.94 1.97 5.98 3.15 3.03 2.51 2.69

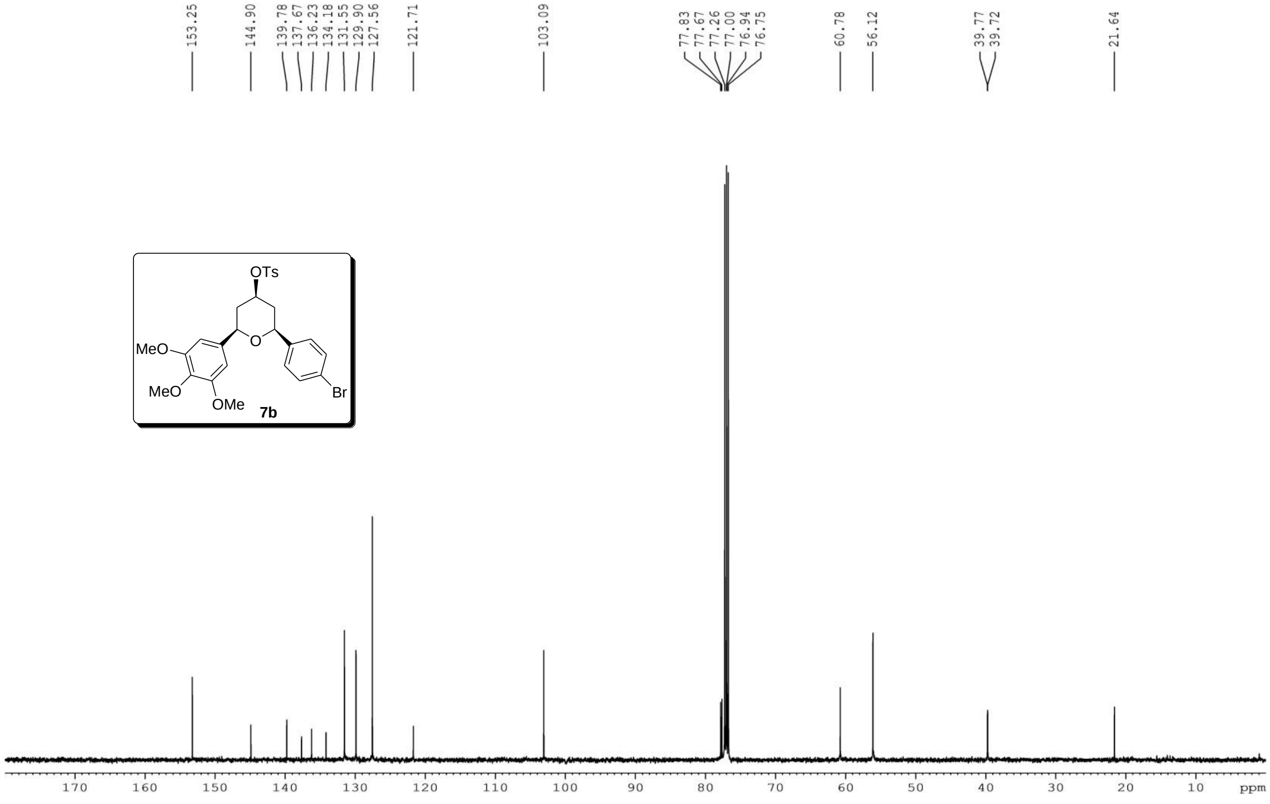
153.25 144.90 139.78 137.67 136.23 134.18 131.55 129.90 127.56 121.71 103.09 77.83 77.67 77.26 77.00 76.94 76.75 60.78 56.12 39.77 39.72 21.64
OTs
MeO
MeO
OMe
O
Br
7b
170 160 150 140 130 120 110 100 90 80 70 60 50 40 30 20 10 ppm

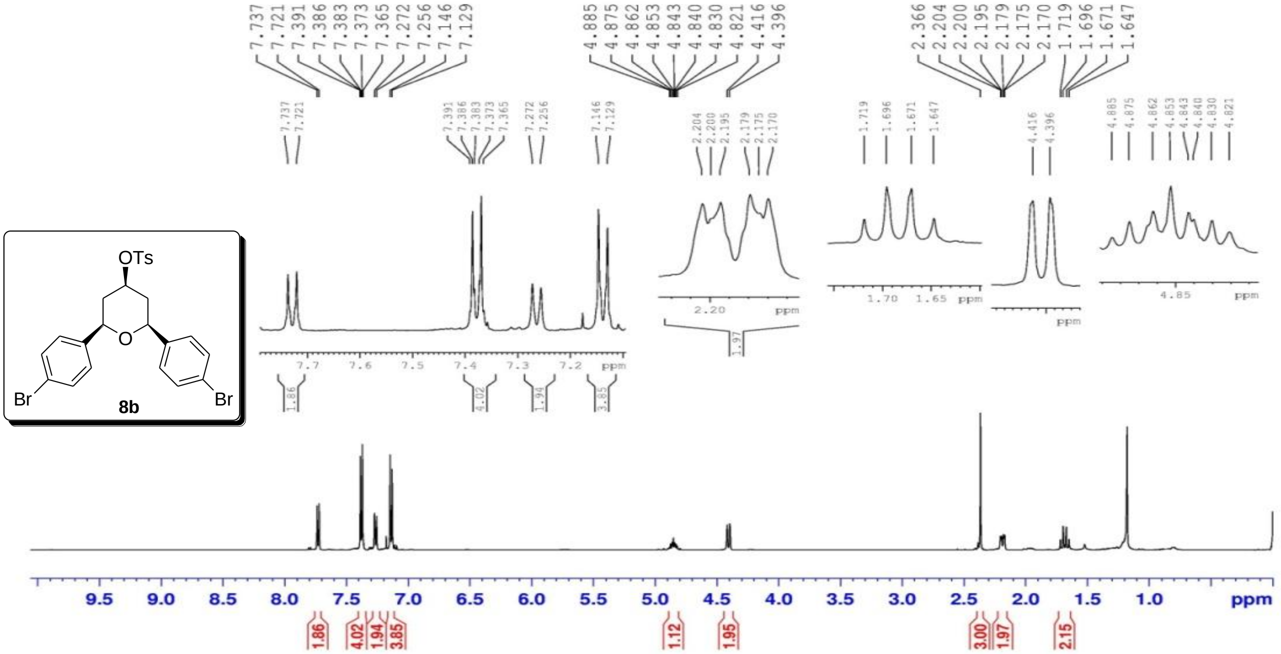
7.737 7.721 7.391 7.386 7.383 7.373 7.365 7.272 7.256 7.146 7.129
4.885 4.875 4.862 4.853 4.843 4.840 4.830 4.821 4.416 4.396
2.366 2.204 2.200 2.195 2.179 2.175 2.170 1.719 1.696 1.671 1.647
OTs
O
Br
Br
8b
1.86 4.02 1.94 3.85 1.12 1.95 3.00 1.97 2.15
ppm

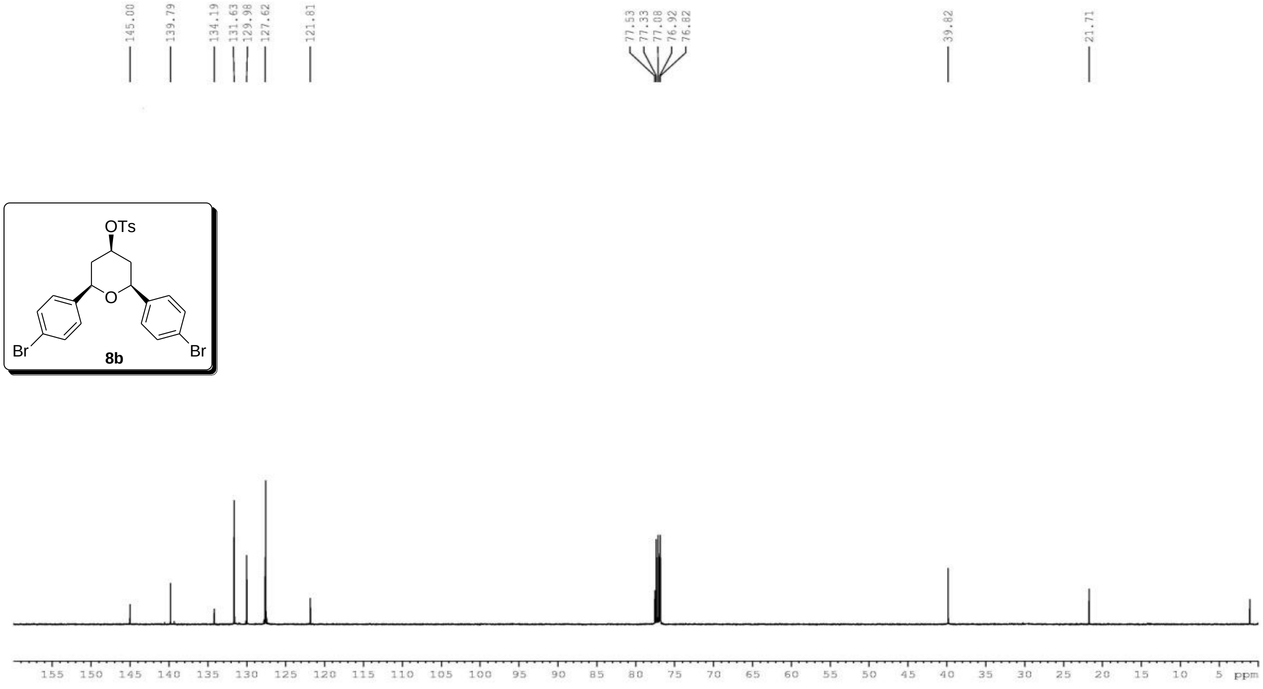
145.00 139.79 134.19 131.63 129.98 127.62 121.81
77.53 77.33 77.08 76.92 76.82
39.82
21.71
OTs
O
Br
Br
8b
ppm

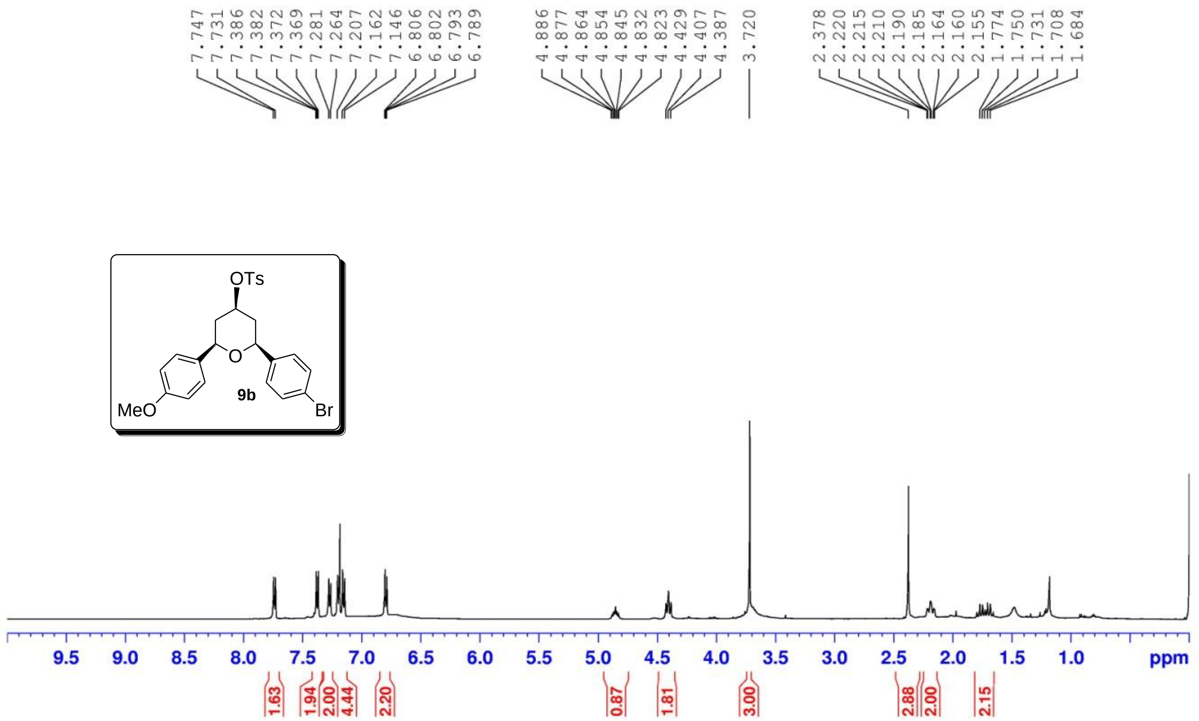

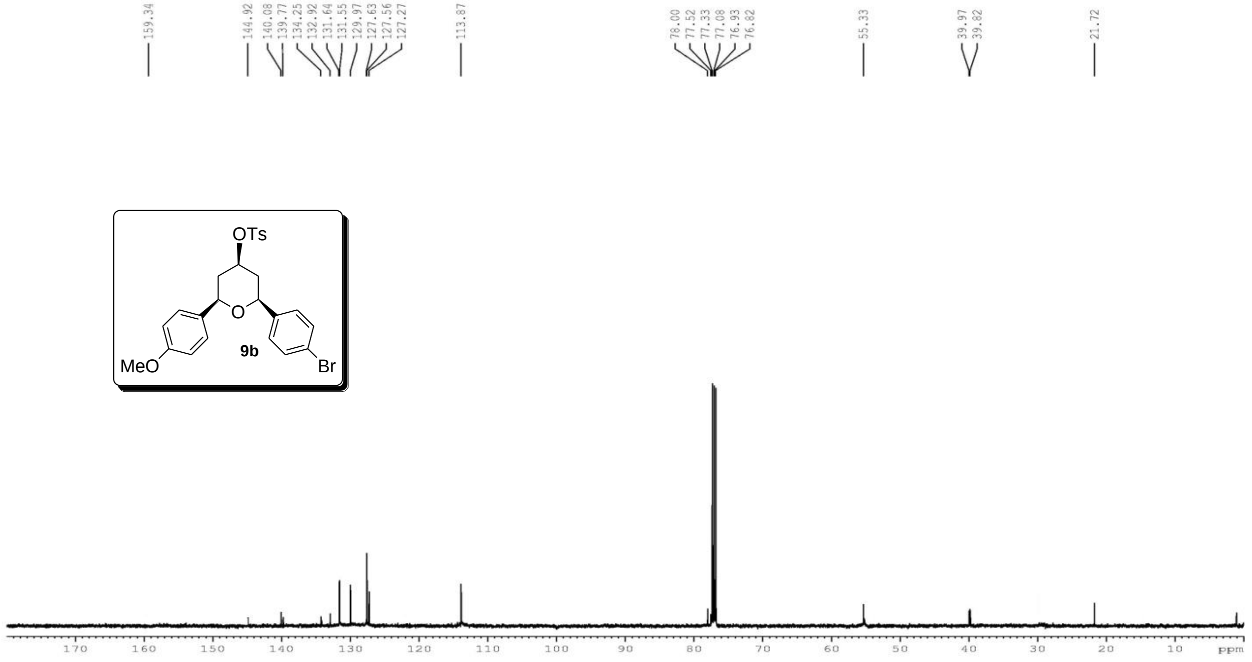

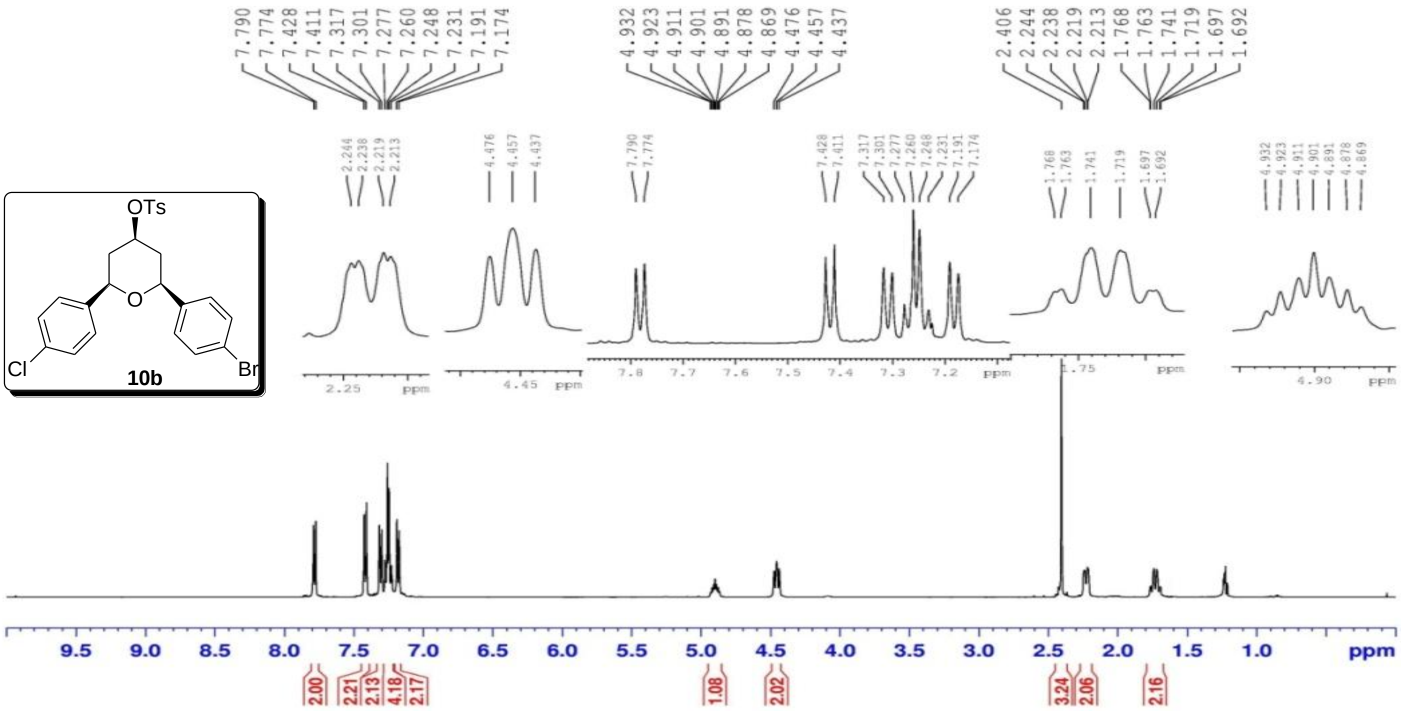
7.790 7.774 7.428 7.411 7.317 7.301 7.277 7.260 7.248 7.231 7.191 7.174
4.932 4.923 4.911 4.901 4.891 4.878 4.869 4.476 4.457 4.437
2.406 2.244 2.238 2.219 2.213 1.768 1.763 1.741 1.719 1.697 1.692
OTs
O
Cl
Br
10b
9.5 9.0 8.5 8.0 7.5 7.0 6.5 6.0 5.5 5.0 4.5 4.0 3.5 3.0 2.5 2.0 1.5 1.0 ppm
2.00 2.21 2.13 4.18 2.17 1.08 2.02 3.24 2.06 2.16

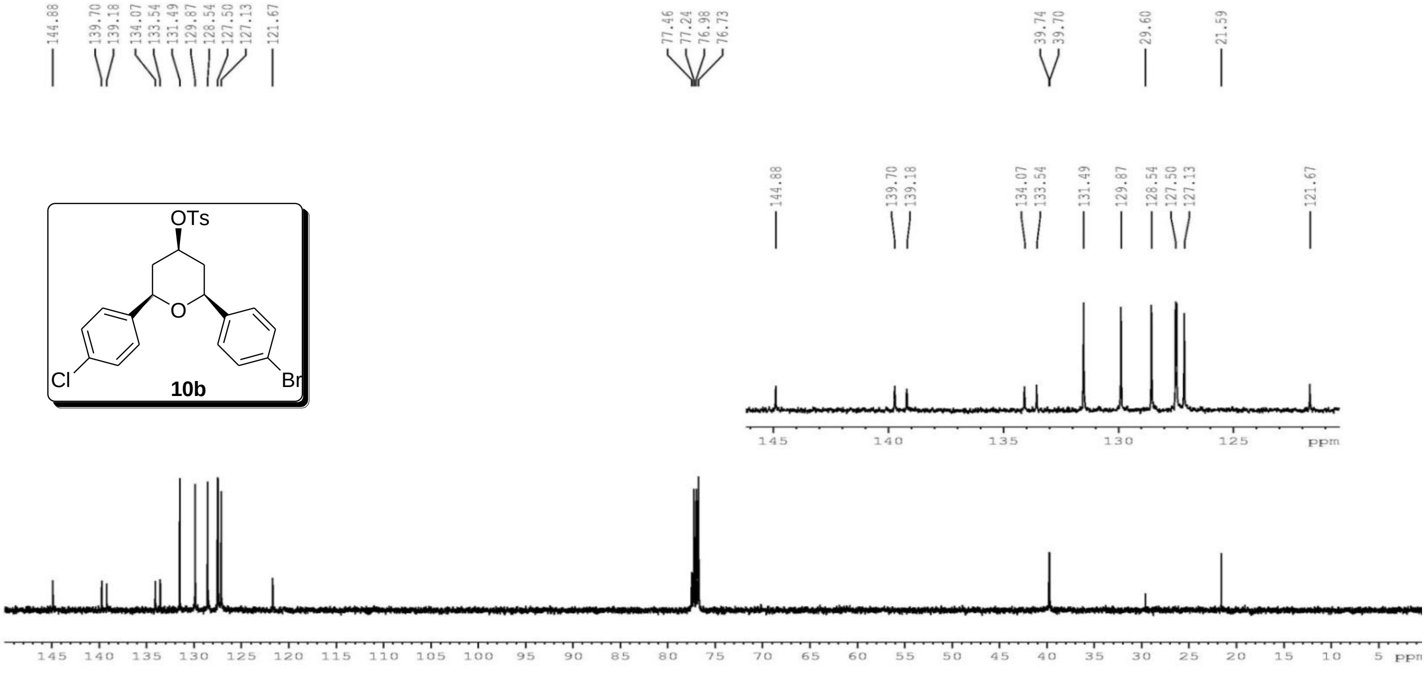
144.88 139.70 139.18 134.07 133.54 131.49 129.87 128.54 127.50 127.13 121.67
77.46 77.24 76.98 76.73
39.74 39.70 29.60 21.59
OTs
O
Cl
Br
10b
145 140 135 130 125 120 115 110 105 100 95 90 85 80 75 70 65 60 55 50 45 40 35 30 25 20 15 10 5 ppm

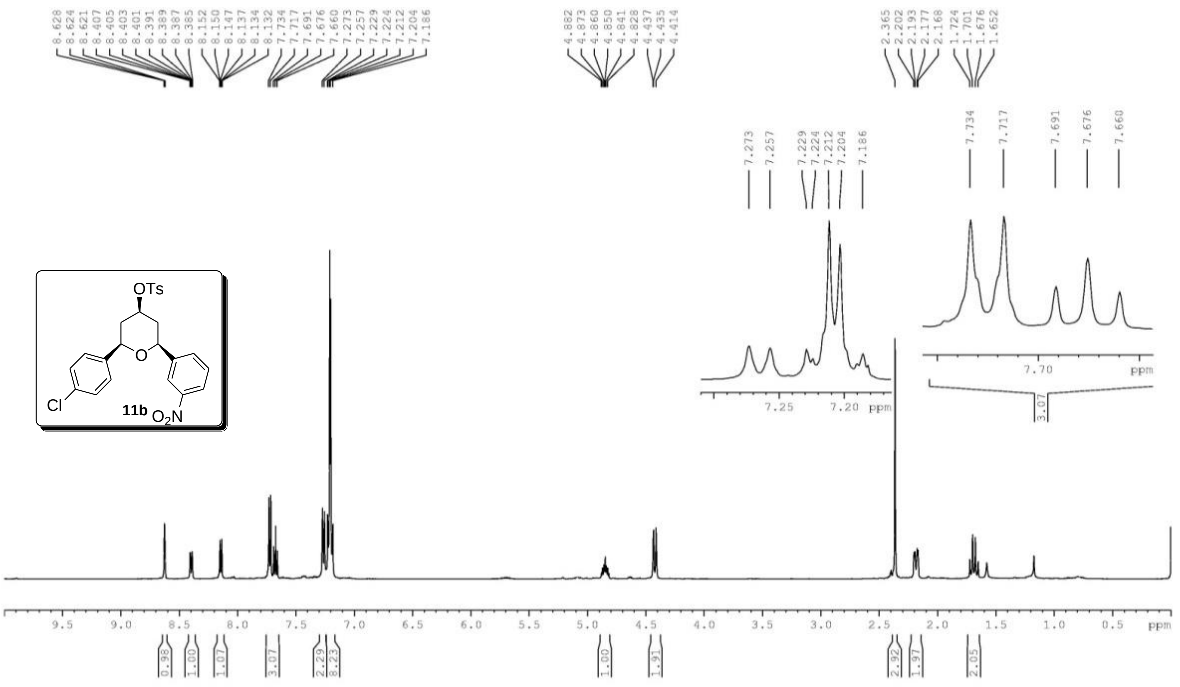

8.628
8.624
8.621
8.407
8.405
8.403
8.401
8.391
8.389
8.387
8.385
8.152
8.150
8.147
8.137
8.134
8.132
7.734
7.717
7.691
7.676
7.660
7.273
7.257
7.229
7.224
7.212
7.204
7.186
4.882
4.873
4.860
4.850
4.841
4.828
4.437
4.435
4.414
2.365
2.202
2.193
2.177
2.168
1.724
1.701
1.676
1.652
OTs
O
Cl
11b
$O_2N$
ppm


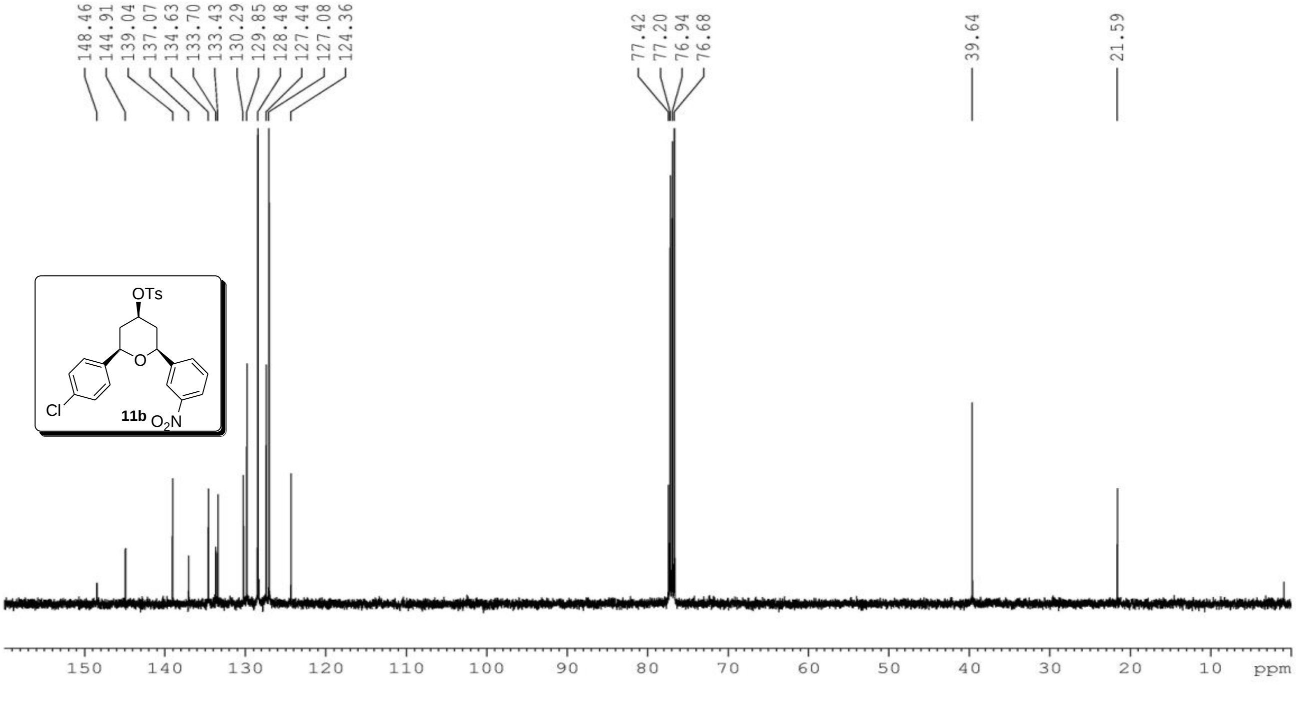

148.46
144.91
139.04
137.07
134.63
133.70
133.43
130.29
129.85
128.48
127.44
127.08
124.36
77.42
77.20
76.94
76.68
39.64
21.59
OTs
O
Cl
11b
$O_2N$
ppm

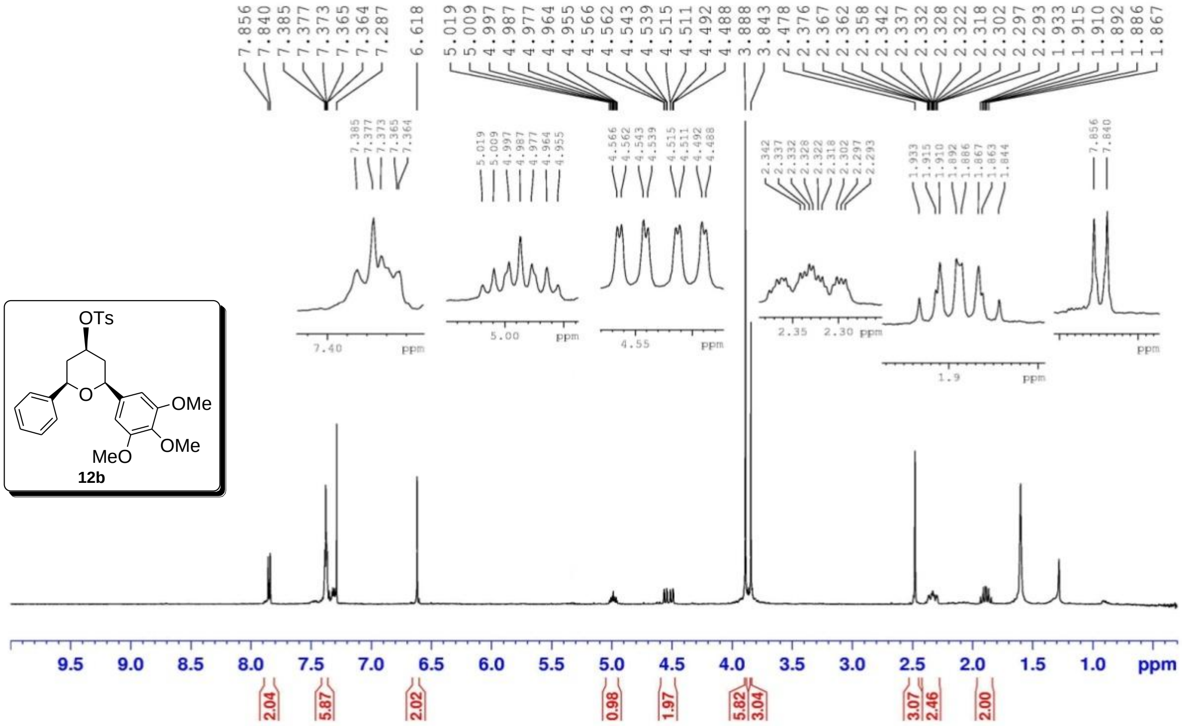
OTs
O
OMe
MeO
OMe
12b
9.5 9.0 8.5 8.0 7.5 7.0 6.5 6.0 5.5 5.0 4.5 4.0 3.5 3.0 2.5 2.0 1.5 1.0 ppm
2.04 5.87 2.02 0.98 1.97 5.82 3.04 3.07 2.46 2.00

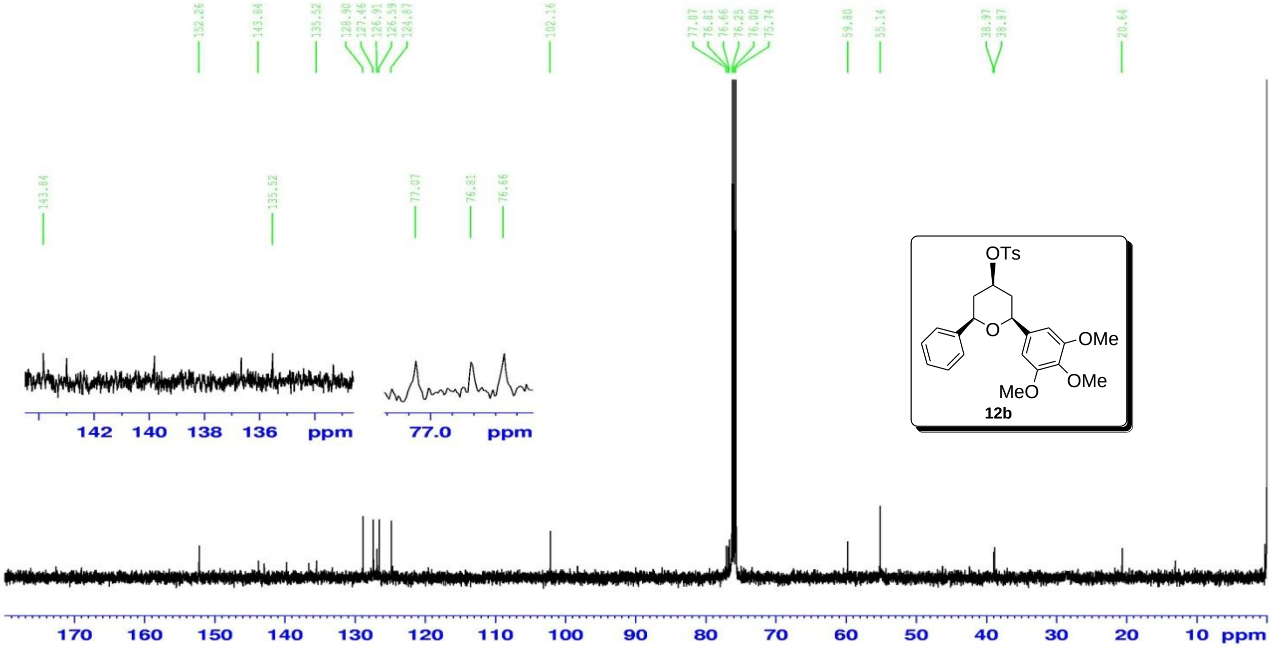
152.26 143.84 135.52 128.90 127.46 126.91 126.59 124.87 102.16 77.07 76.81 76.66 76.25 76.00 75.74 59.80 55.14 38.97 38.87 20.64
OTs
O
OMe
MeO
OMe
12b
170 160 150 140 130 120 110 100 90 80 70 60 50 40 30 20 10 ppm

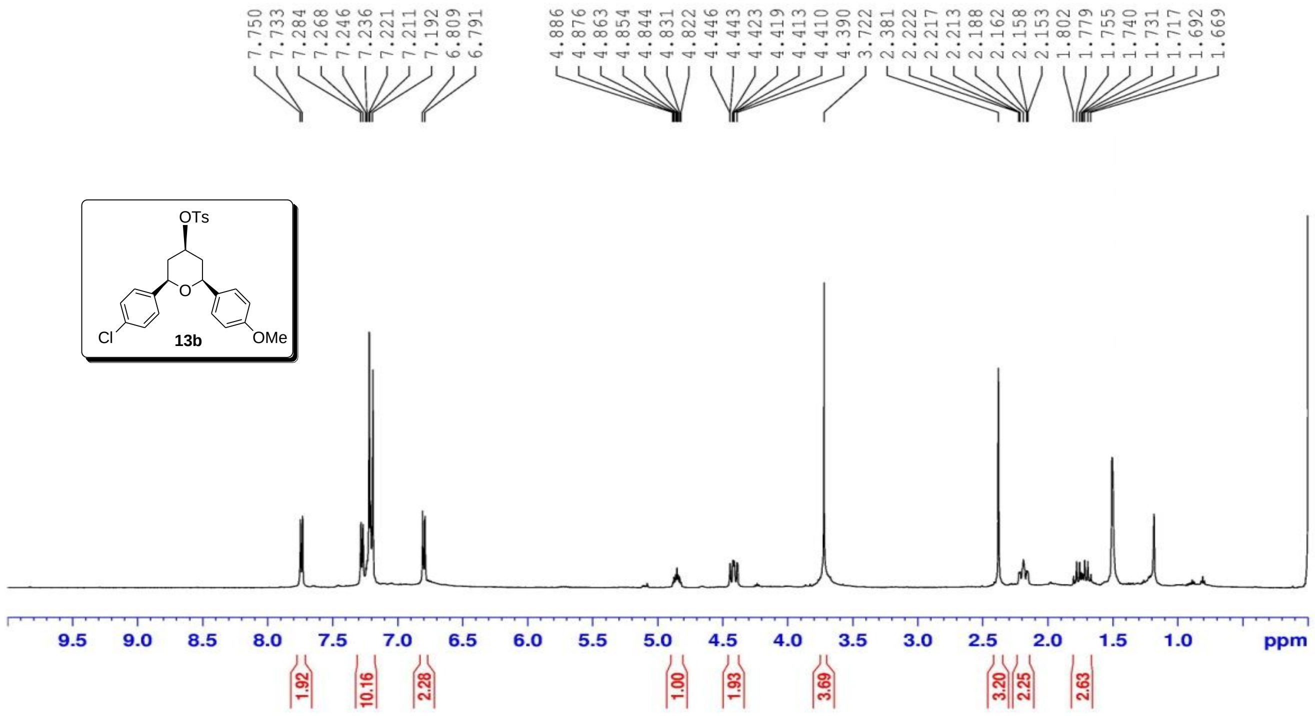
7.750 7.733 7.284 7.268 7.246 7.236 7.221 7.211 7.192 6.809 6.791
4.886 4.876 4.863 4.854 4.844 4.831 4.822 4.446 4.443 4.423 4.419 4.413 4.410 4.390 3.722 2.381 2.222 2.217 2.213 2.188 2.162 2.158 2.153 1.802 1.779 1.755 1.740 1.731 1.717 1.692 1.669
OTs
O
Cl
13b
OMe
9.5 9.0 8.5 8.0 7.5 7.0 6.5 6.0 5.5 5.0 4.5 4.0 3.5 3.0 2.5 2.0 1.5 1.0 ppm
1.92 10.16 2.28 1.00 1.93 3.69 3.20 2.25 2.63

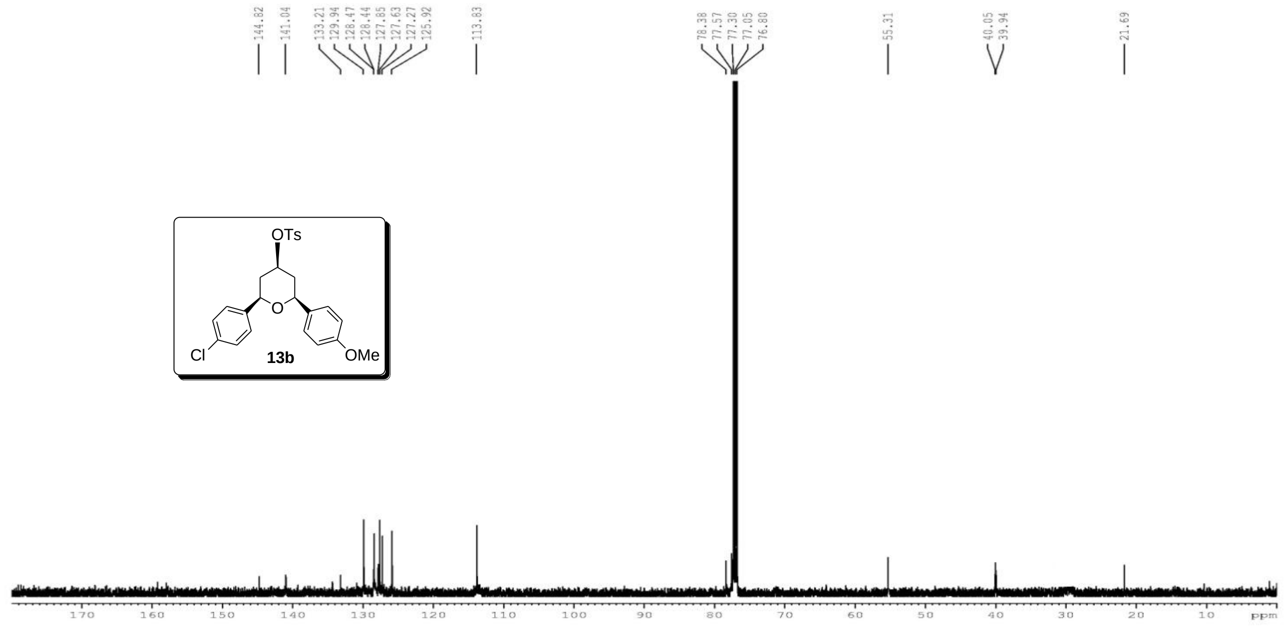
144.82 141.04 133.21 129.94 128.47 128.44 127.85 127.63 127.27 125.92 113.83
78.38 77.57 77.30 77.05 76.80 55.31 40.05 39.94 21.69
OTs
O
Cl
13b
OMe
170 160 150 140 130 120 110 100 90 80 70 60 50 40 30 20 10 ppm

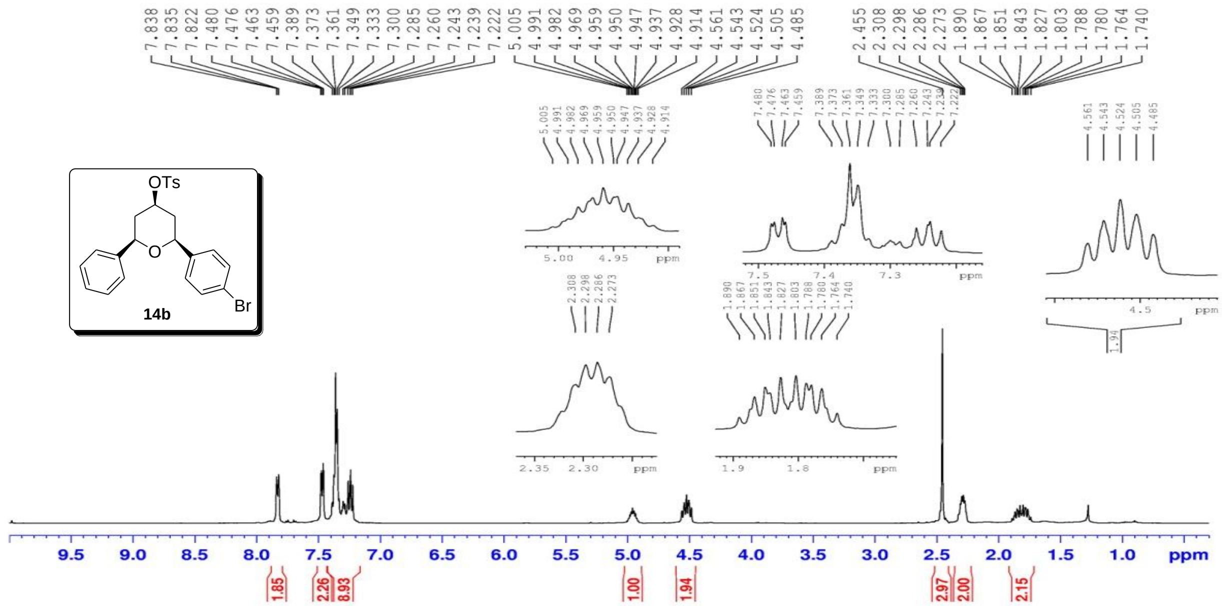

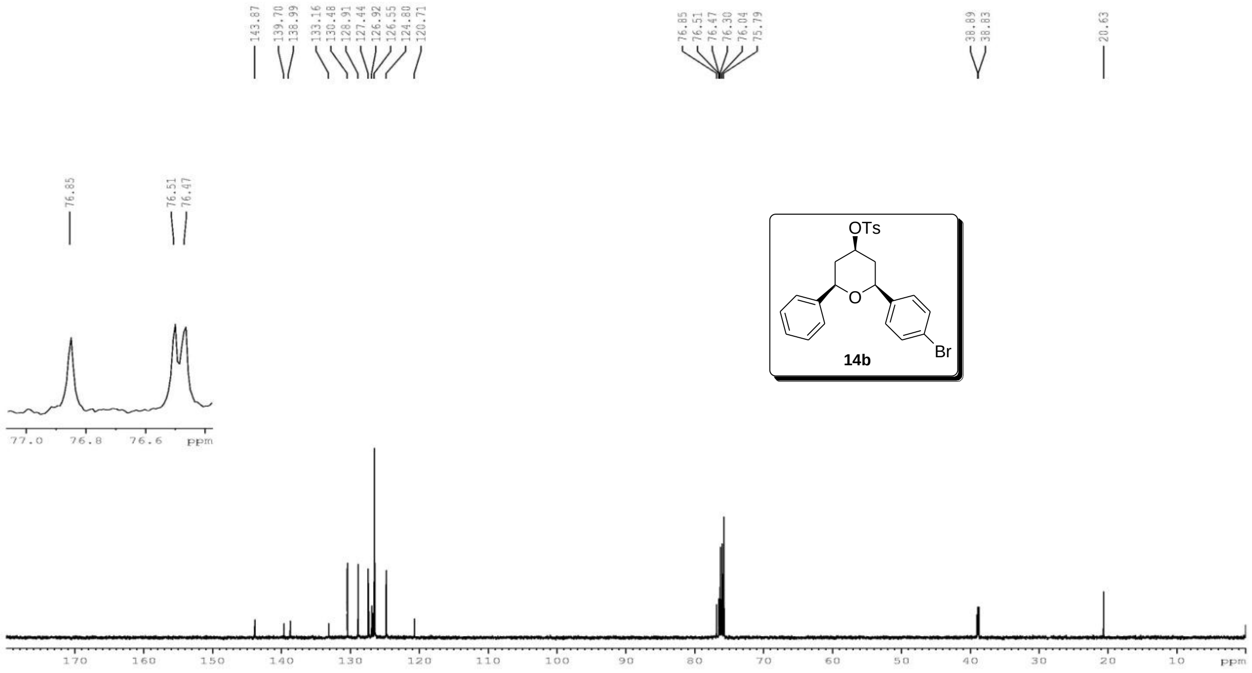

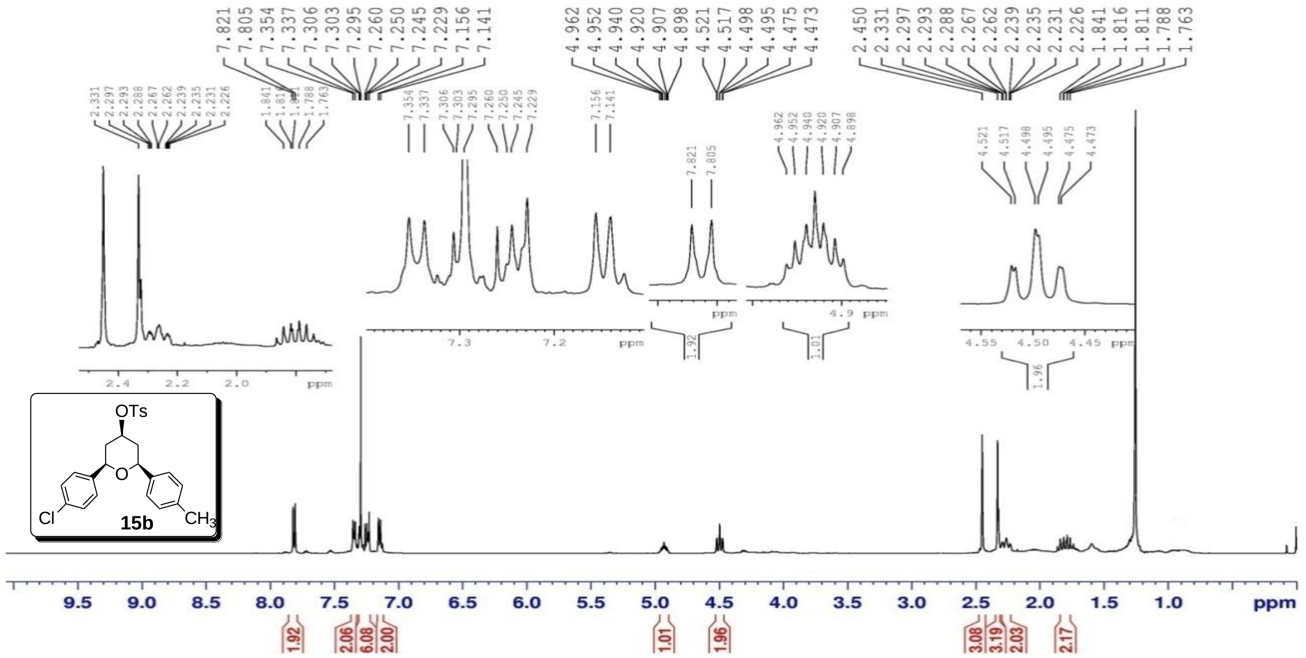
7.821
7.805
7.354
7.337
7.306
7.303
7.295
7.260
7.250
7.245
7.229
7.156
7.141
4.962
4.952
4.940
4.920
4.907
4.898
4.521
4.517
4.498
4.495
4.475
4.473
2.450
2.331
2.297
2.293
2.288
2.267
2.262
2.239
2.235
2.231
2.226
1.841
1.816
1.811
1.788
1.763
OTs
O
Cl
CH3
15b
1.92
2.06
6.08
2.00
1.01
1.96
3.08
3.19
2.03
2.17
ppm

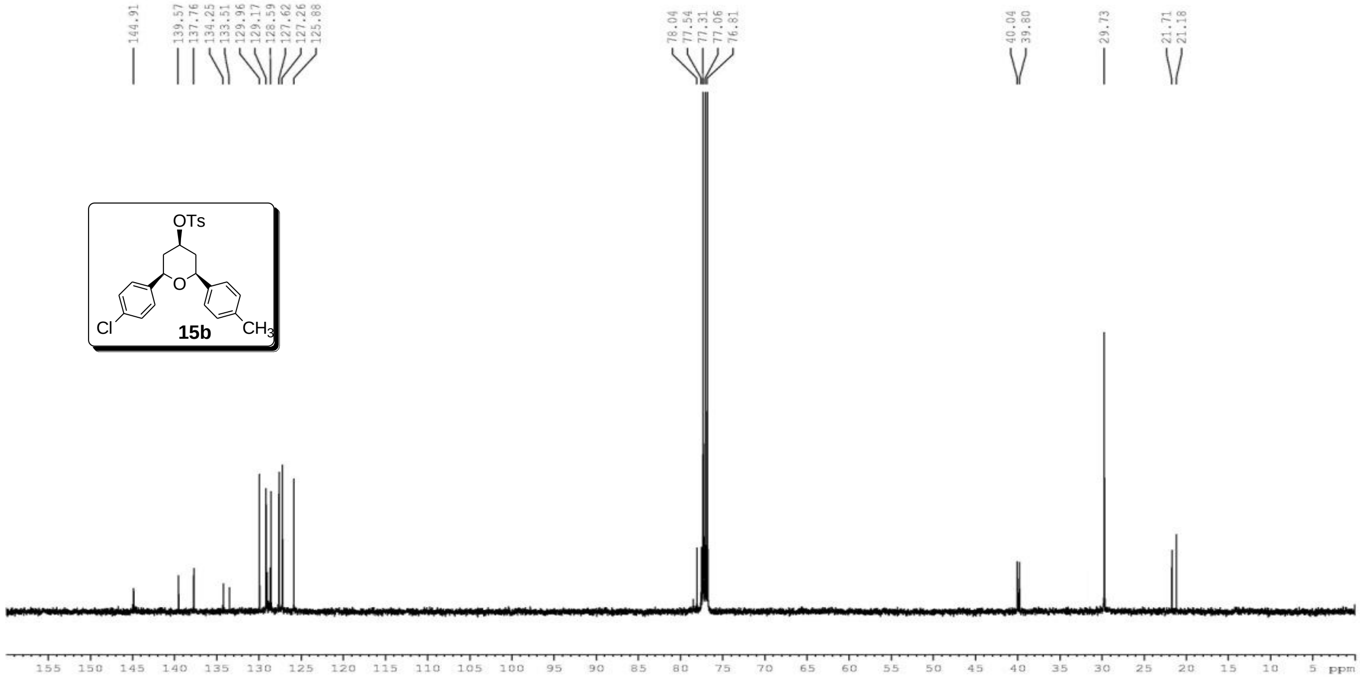
144.91
139.57
137.76
134.25
133.51
129.96
129.17
128.59
127.62
127.26
125.88
78.04
77.54
77.31
77.06
76.81
40.04
39.80
29.73
21.71
21.18
OTs
O
Cl
CH3
15b
ppm

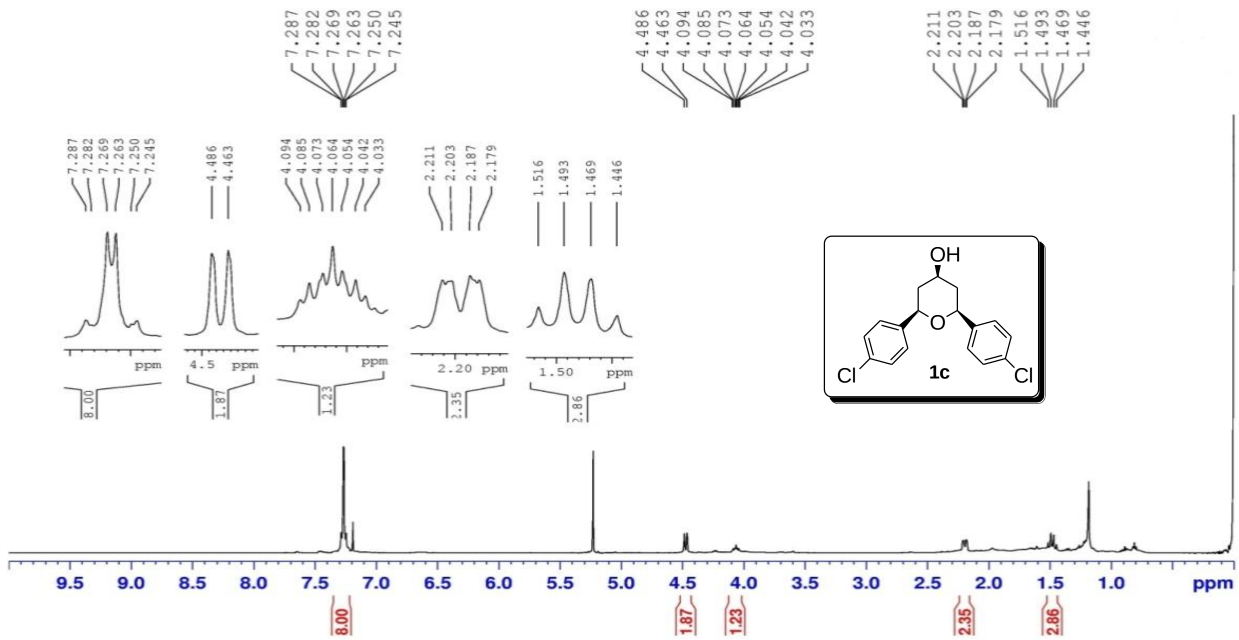
7.287
7.282
7.269
7.263
7.250
7.245
4.486
4.463
4.094
4.085
4.073
4.064
4.054
4.042
4.033
2.211
2.203
2.187
2.179
1.516
1.493
1.469
1.446
OH
O
Cl
1c
Cl
8.00
1.87
1.23
2.35
2.86
ppm

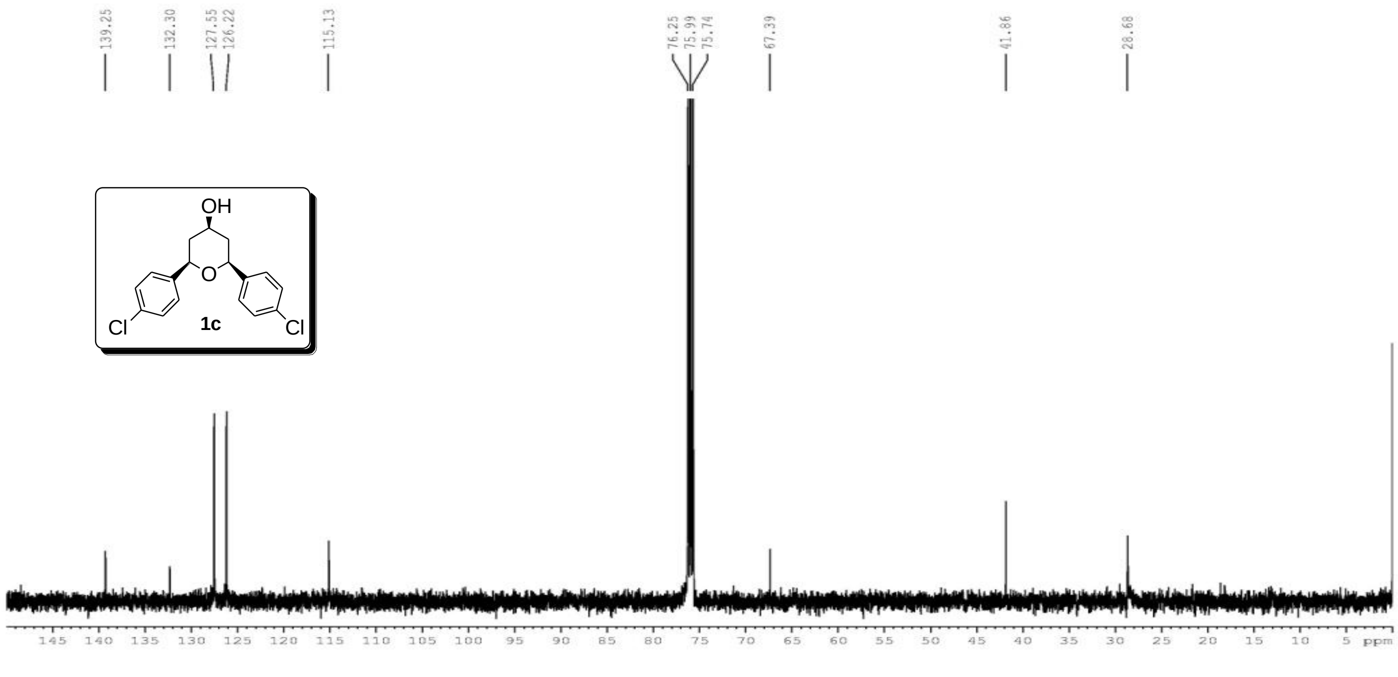
139.25
132.30
127.55
126.22
115.13
76.25
75.99
75.74
67.39
41.86
28.68
OH
O
Cl
1c
Cl
ppm

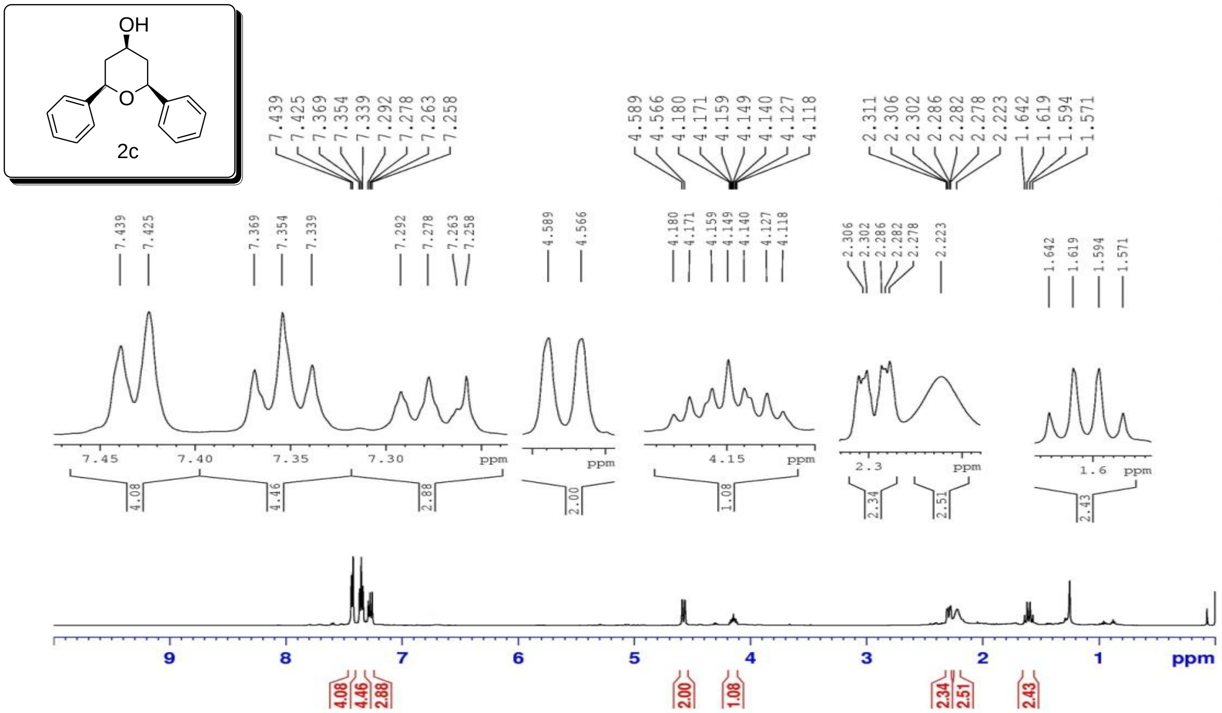
OH
O
2c
7.439 7.425 7.369 7.354 7.339 7.292 7.278 7.263 7.258
4.589 4.566 4.180 4.171 4.159 4.149 4.140 4.127 4.118
2.311 2.306 2.302 2.286 2.282 2.278 2.223 1.642 1.619 1.594 1.571
4.08 4.46 2.88 2.00 1.08 2.34 2.51 2.43
ppm

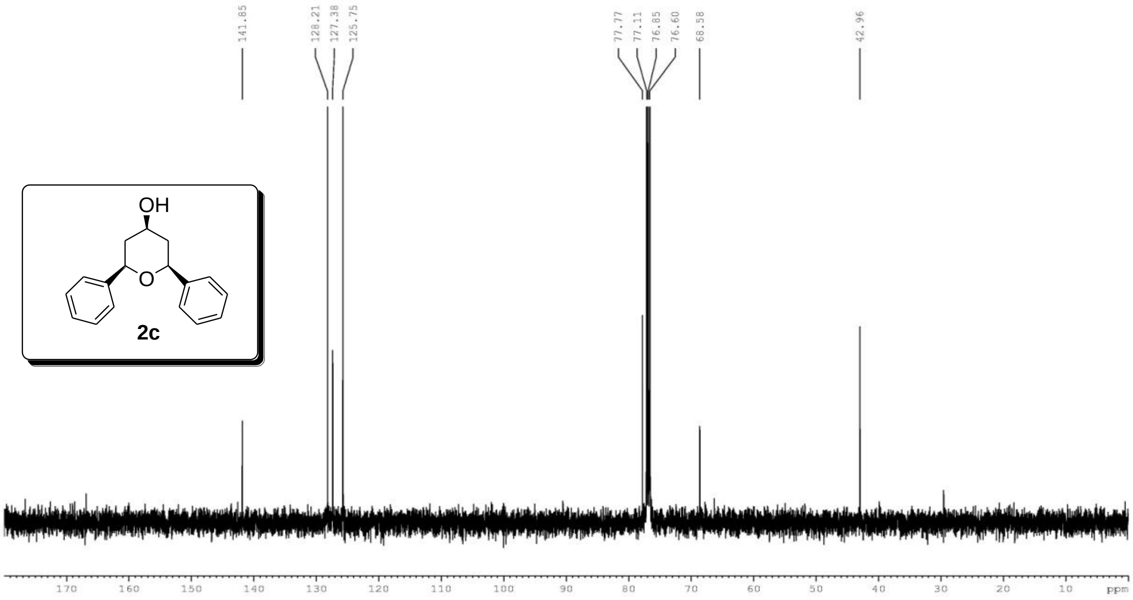
141.85
128.21 127.38 125.75
77.77 77.11 76.85 76.60 68.58
42.96
OH
O
2c
ppm

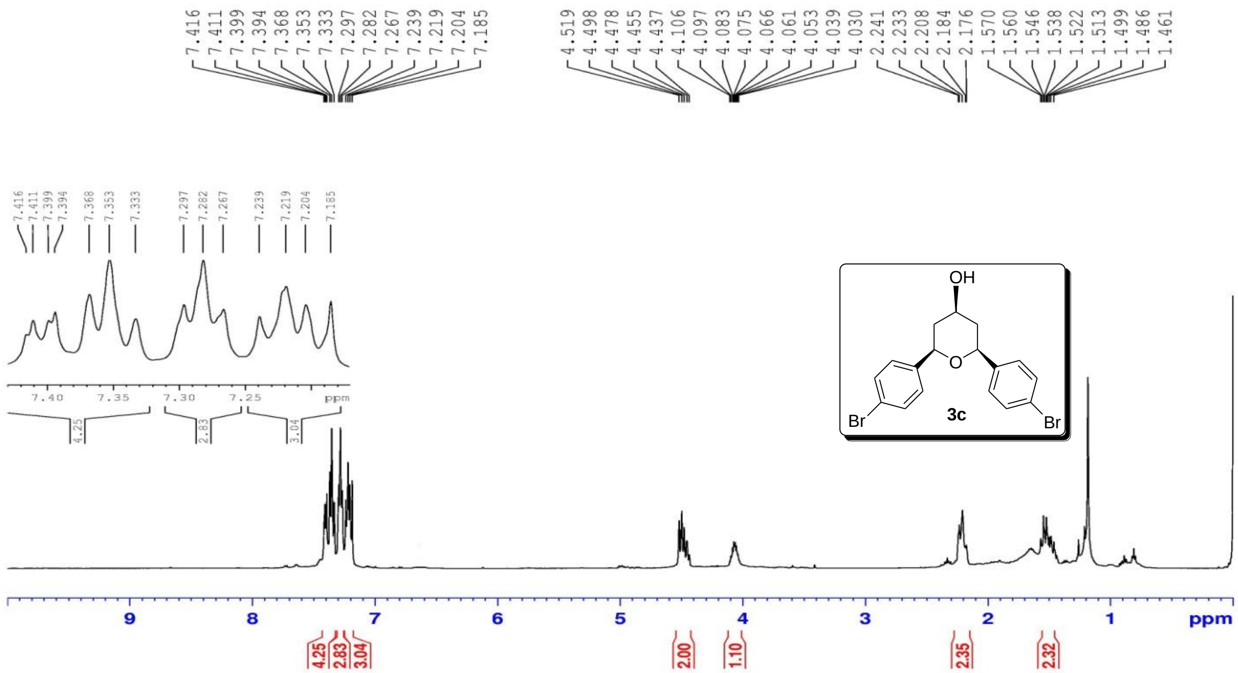
7.416
7.411
7.399
7.394
7.368
7.353
7.333
7.297
7.282
7.267
7.239
7.219
7.204
7.185
4.519
4.498
4.478
4.455
4.437
4.106
4.097
4.083
4.075
4.066
4.061
4.053
4.039
4.030
2.241
2.233
2.208
2.184
2.176
1.570
1.560
1.546
1.538
1.522
1.513
1.499
1.486
1.461
OH
O
Br
3c
Br
4.25
2.83
3.04
2.00
1.10
2.35
2.32
ppm

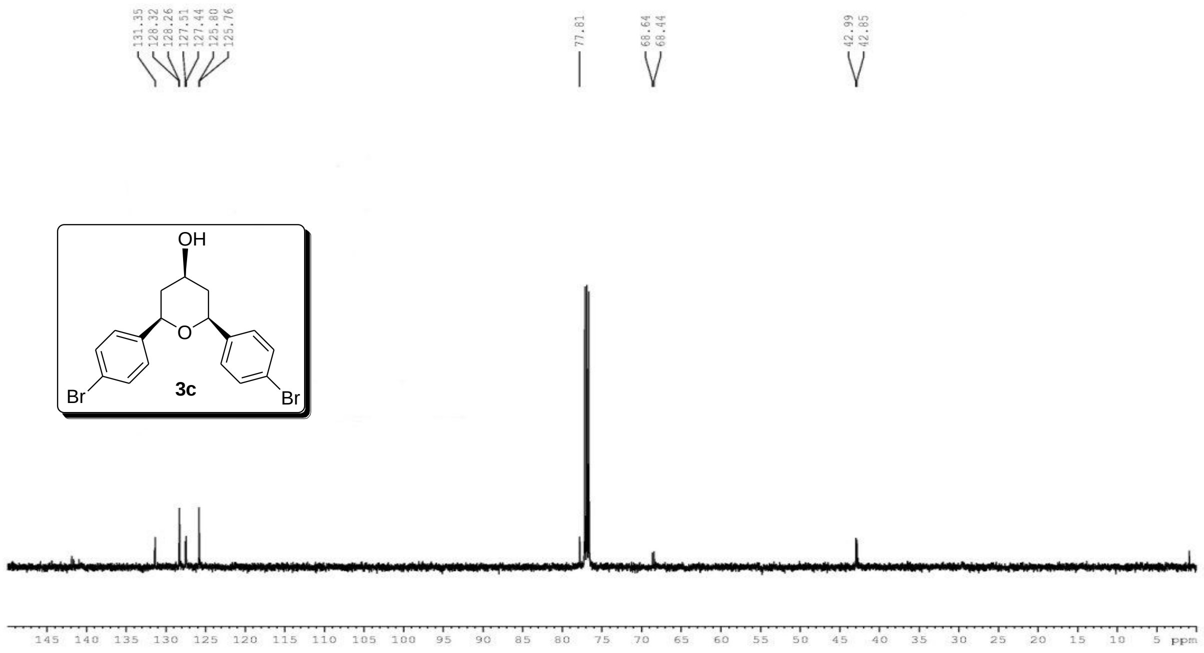
131.35
128.32
128.26
127.51
127.44
125.80
125.76
77.81
68.64
68.44
42.99
42.85
OH
O
Br
3c
Br
ppm
